# Implementing robotic-assisted therapy in stroke rehabilitation: a structured narrative review of evidence and technology adoption barriers in Indonesia

**DOI:** 10.3389/fresc.2026.1942904

**Published:** 2026-10-08

**Authors:** Ezra B. Wijaya, Phyo-Wai Thu, Pao-Chu Chen

**Affiliations:** 1 Department of Physiotherapy, Faculty of Health Science and Technology, Binawan University, Jakarta, Indonesia; 2 Hualien Tzu Chi Hospital, Buddhist Tzu Chi Medical Foundation, Hualien, Taiwan; 3 School of Medicine, College of Medicine, Tzu Chi University, Hualien, Taiwan

**Keywords:** biofeedback, implementation barriers, Indonesia, recovery of function, robotic-assisted therapy, stroke rehabilitation

## Abstract

**Introduction:**

Robotic-assisted therapy (RAT) has emerged as a promising intervention for post-stroke recovery; however, its implementation in developing countries such as Indonesia remains limited by economic, technical, and systemic barriers. This structured narrative review summarizes clinical effect reported by systematics review and meta-analyses and examines implementation consideration and potential economic feasibility within the Indonesian healthcare context.

**Method:**

A structured review of systematic reviews and meta-analyses (2015–2025) was conducted using PubMed, Scopus, PEDro, and Cochrane Library. Studies involving adult with stroke were included. Findings were synthesized narratively because of heterogeneity. Implementation barriers were analyzed, and a scenario-based economic model was developed to assess feasibility of RAT in Indonesia. QALY-equivalent outcomes (QEOs) were estimated using ratio-based value-transfer approach that scaled published reference values according to observed functional effects.

**Result:**

Evidence from 22 systematic reviews indicated that RAT improved functional outcomes compared with conventional therapy, particularly gait and upper-limb function (effect sizes: 0.02–0.52). Key implementation barriers included limited device availability, inadequate workforce training, and regulatory constraints. Modelled ICERs ranged from US$236.23–752.31 per QEO for upper-limb rehabilitation and US$558.86–4,890 per QEO for gait-related rehabilitation. Most estimates remained below the Indonesian willingness-to-pay threshold (<US$5,030), suggesting a favourable economic profile under model assumptions, particularly in higher-resource provinces, while lower-income regions may face affordability constraints.

**Conclusion:**

RAT shows potential clinical benefits, but implementation in Indonesia should consider clinical effectiveness, economic feasibility, infrastructure, workforce, technology acceptance, and reimbursement. A phased approach in suitable tertiary-care settings may support wider adoption based on local evidence.

## 1 Introduction

Stroke is one of the major public health issues in Indonesia. The Global Burden of Disease (GBD) 2019 study reported 642,943 new stroke cases and 4,9 million total cases annually, with an age-standardized incidence of 293.3 per 100,000 population in Indonesia and projections suggesting further increases by 2050 (1, 2). Moreover, stroke imposes substantial economic costs. Data from the Government Bureau of Statistics Indonesia in 2023 shows that stroke was one of the three most expensive chronic diseases, accounting for over 3.4 million cases and 18.5% of all death in Indonesia (3, 4). This elevated mortality and incidence underscores the urgent need for effective preventive strategies, timely medical interventions, and comprehensive rehabilitation programs designed for the Indonesian population (5).

Stroke occurs when cerebral blood flow is interrupted, either due to ischemia or vascular rupture, which lead to acute neurological damage and functional impairments (6). These impairments often manifest as motor deficits, cognitive decline, and language disorders. Consequently, post-stroke rehabilitation plays a critical role in promoting functional recovery by facilitating neuroplastic reorganization through intensive, task-specific, and repetitive practice training (7).

Current post-stroke rehabilitation strategies such as task-specific training have been shown to improve motor function, balance, gait velocity, and activities of daily living more effectively than traditional approaches (8). Moreover, evidence indicates that task-specific training performed at moderate to high intensities results in greater gait recovery compared with lower-intensity training. Some studies also highlight that dose specific training including the number of repetitions, frequency, and intensity is a key factor in maximizing the treatment outcomes after stroke (9).

Although few comprehensive randomized controlled trials have investigated robotic rehabilitation, an increasing number of small clinical studies and case reports have examined robotic-assisted therapy in both the subacute and chronic phases following stroke (10, 11). For example, wearable overground exoskeletons on the market have opened new opportunities for rehabilitation by offering high-volume, task-oriented practice, which has been shown to support recovery in individuals at both early and later stages of post-stroke rehabilitation (11).

Robotic assisted therapy plays a central role in gait rehabilitation for individuals recovering from stroke. During overground walking, these devices assist with gait initiation and limb advancement, providing either full or partial support. The exoskeleton's framework also improves stability and balance, promoting safer and more effective walking practice (12). Similarly, in upper limb rehabilitation, these technologies, when combined with conventional therapy, provide task-specific, repetitive grasping and object manipulation using the fingers, which eventually helps retain motor patterns and enhances neuroplasticity (13, 14).

Despite the promising clinical benefits of robotic assisted therapy in stroke rehabilitation, the clinical adoption of RAT in clinical and hospital remains limited. A study in the Italian healthcare community reported several barriers such, including high costs, lack of scientific evidence, limited professional expertise, and lack of awareness about rehabilitation devices hinder broader adoption (15). Another study evaluating the cost-effectiveness of robotic assisted therapy in Singapore found that, while the technology offers potential benefits, its high cost poses a significant barrier to implementation in routine clinical practice (16).

Most evidence on robotic-assisted therapy (RAT) for stroke rehabilitation have been conducted in high-income countries, making it difficult to directly apply these findings to low- and middle-income countries such as Indonesia. The literature also varies substantially in intervention protocols, rehabilitation intensity, outcome measures, and healthcare settings. Because of this heterogeneity, a structured narrative review was considered more appropriate than a formal umbrella review or health technology assessment. Therefore, assessing RAT within the specific healthcare, economic, and implementation context of a developing country is necessary to determine how established clinical evidence may translate into feasible technology adoption.

Indonesia is not presented simply as another resource-limited setting. Its implementation environment is shaped by substantial geographic and administrative decentralization, variation in hospital service capacity (17–19), regional economic inequality and differences in healthcare access (3), and national healthcare and medical-device regulatory arrangements (20). These interacting conditions may create substantial differences in the feasibility of RAT adoption across healthcare settings within the same country, making Indonesia a relevant context for examining how established clinical evidence can be translated into technology adoption.

To address these gaps, this narrative review focuses on three domains. First, it summarizes clinical effect estimates reported by systematic reviews and meta-analyses to characterize the magnitude of clinical benefits of robotic-assisted therapy (RAT) compared with conventional therapy across functional outcomes. Second, it examines clinical and economic implications by integrating Indonesian health financing data into a model-based cost-effectiveness analysis to evaluate feasibility within a resource-limited setting. Third, it provides a structured narrative synthesis of implementation perspectives, including clinical practice integration, barriers to adoption, rehabilitation infrastructure, and industry collaboration, to provide a conceptual context for considering RAT adoption within the Indonesian healthcare context.

Taken together, this review integrates evidence from clinical effectiveness synthesis, country-specific economic modelling, and implementation analysis to provide a comprehensive overview of robotic-assisted stroke rehabilitation within the context of a developing healthcare system.

## 2 Method

### 2.1 Study design

This study was designed as a structured narrative review integrating evidence synthesis, implementation analysis, and exploratory economic modelling related to robotic-assisted therapy (RAT) in stroke rehabilitation. PRISMA-based reporting principles were applied to improve transparency in literature identification and study selection (Figure 1); however, the study was not intended as a formal umbrella review or quantitative meta-analysis. Given the substantial heterogeneity in intervention protocols, robotic systems, rehabilitation settings, and outcome measures across the included literature, a narrative synthesis approach was considered more appropriate for contextual interpretation and policy-oriented discussion.

**Figure 1:**
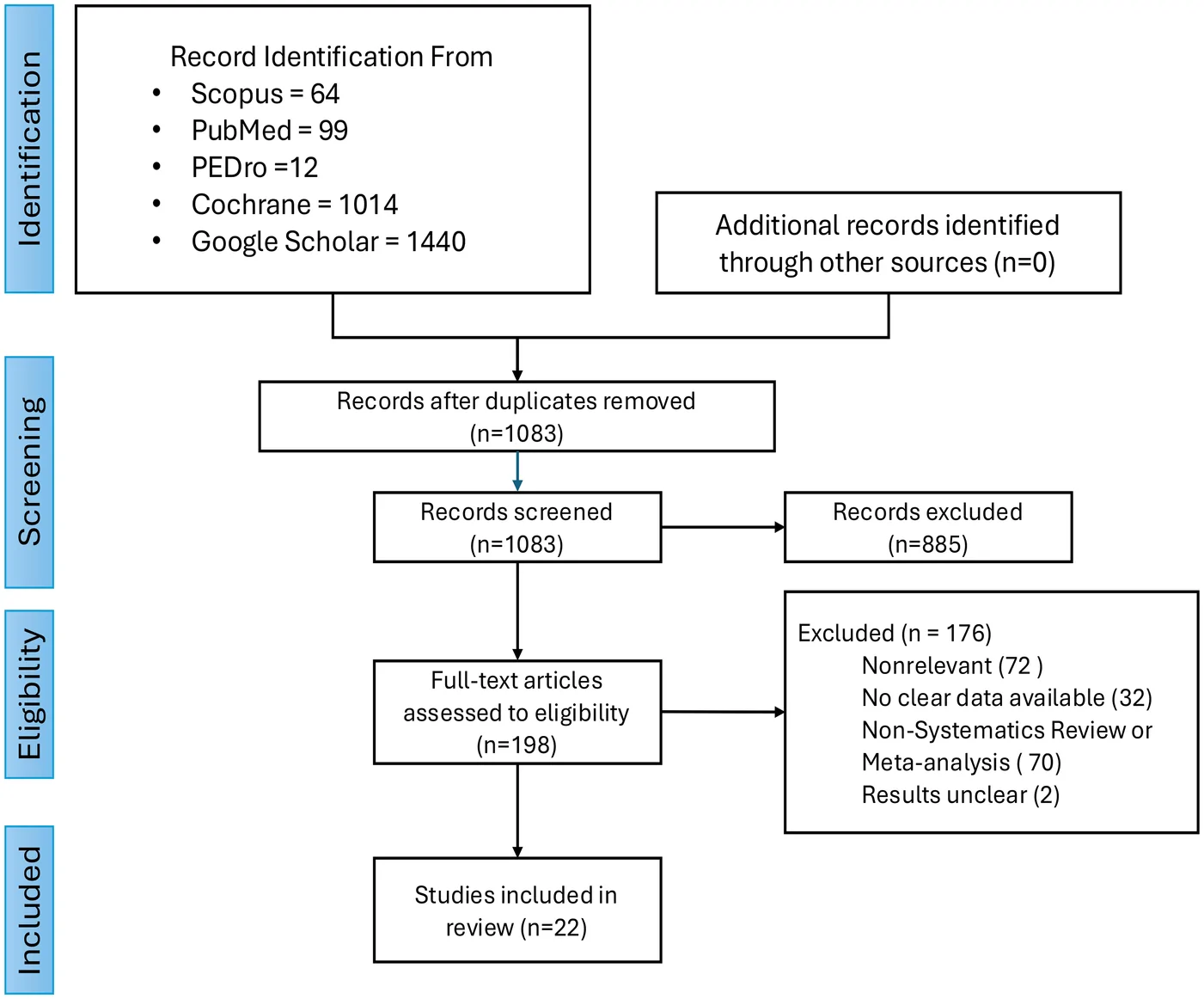
Flow diagram of the literature selection process across review phases.

### 2.2 Literature search

A comprehensive literature search was conducted in PubMed, Scopus, the Physiotherapy Evidence Database (PEDro), the Cochrane Library, and Google Scholar to identify relevant studies published between 2015 and 2025. The search strategy was designed to capture evidence on robotic-assisted therapy (RAT) in stroke rehabilitation across clinical, technological, and implementation domains.

Search terms were developed using combinations of keywords and Medical Subject Headings (MeSH) where applicable, including: “stroke AND robotics AND active,” “stroke AND gait AND HAL,” “stroke AND robotics AND Hybrid Assistive Limb,” “stroke AND exoskeleton AND biofeedback,” “stroke AND robotics AND exoskeleton,” and “stroke AND robotics AND inpatient.” Boolean operators (AND/OR) were used to optimize sensitivity and specificity of the search.

### 2.3 Study selection and data extraction

Study selection, screening, and data extraction were conducted independently by two reviewers. Any discrepancies regarding study eligibility, classification, or extracted data were resolved through discussion and consensus. For extracted characteristics and classifications reported in the tables and Supplementary File, including treatment type, session duration, and outcome-measure classification, the two reviewers cross-checked and discussed the extracted information. When consensus could not be readily reached, the issue was discussed with the third author to reach a final consensus. Cohen's kappa was not calculated.

This review included systematic reviews investigating robotic-assisted therapy (RAT) in stroke rehabilitation, including those incorporating meta-analyses, to enable structured narrative synthesis of heterogeneous evidence. The focus was on descriptive and contextual interpretation of findings relevant to clinical effectiveness, implementation, and policy considerations, rather than quantitative pooling across reviews.

Eligibility criteria were as follows: (i) included adult stroke populations (≥18 years) with ischemic or haemorrhagic stroke across acute, subacute, or chronic phases; (ii) evaluated robotic-assisted rehabilitation interventions incorporating any form of biofeedback, including visual, auditory, electromyography (EMG)-triggered, or sensor-based feedback systems; and (iii) compared robotic-assisted therapy with conventional physiotherapy or other active rehabilitation approaches. Studies were excluded if they were non-English publications, editorials, conference abstracts, studies without accessible full text, or involved non-stroke populations.

Outcomes of interest included functional mobility, gait performance, balance, activities of daily living (ADL), motor recovery, and feasibility or implementation-related outcomes. Data extraction focused on intervention characteristics, including robotic device type, treatment frequency, session duration, total intervention period, stroke type, and baseline functional status. Additional details on biofeedback modality and whether feedback was delivered in real time or integrated into robotic control systems were extracted when available.

As this study synthesized evidence from previously published systematic reviews and meta-analyses, all extracted data represented pooled or summarized estimates reported within each review rather than individual patient-level data. Extracted findings were synthesized using thematic categorization based on rehabilitation domain, intervention characteristics, implementation barriers, biofeedback integration, and functional outcomes, with emphasis on identifying recurring clinical and contextual patterns relevant to implementation in the Indonesian context.

### 2.4 Economic evaluation and modelling approach

This section presents an exploratory, model-based economic evaluation of robotic-assisted rehabilitation compared with conventional physiotherapy within the Indonesian healthcare context. The analysis was designed as a feasibility-oriented and policy-relevant approximation rather than a full health technology assessment (HTA) or standard cost-utility analysis. The model combines estimated treatment cost and pooled clinical effectiveness, national health insurance (BPJS) reimbursement consideration, and provider-side cost estimates to explore potential economic value and affordability of robotic rehabilitation technologies under the Indonesia healthcare context. Detailed model parameters, assumptions, equations, and calculation procedures are provided in Supplementary File S1.

Clinical effectiveness inputs were derived from pooled effect estimates mean differences (MDs) reported in systematic reviews and meta-analyses comparing RAT with conventional rehabilitation. Because direct utility measures, such as EQ-5D or SF-6D, were unavailable in the included studies, **QALY-equivalent outcomes (QEOs)** were estimated using a ratio-based value-transfer approach adapted from Shankar et al. QEOs represent scaled utility-based outcome estimates derived from published reference values and should not be interpreted as directly measured QALYs. This approach was applied to facilitate comparative economic interpretation while explicitly acknowledging the methodological limitations of utility extrapolation from functional rehabilitation outcomes.

The modelling framework followed the general structure proposed by Shankar et al. (16), integrating pooled clinical effectiveness estimates with treatment cost, Indonesian willingness-to-pay thresholds, BPJS insurance reimbursement consideration, and affordability assumptions. In simplified terms, the model compares the additional cost of robotic-assisted rehabilitation with the additional clinical benefit expressed as QEOs, producing an incremental cost-effectiveness ratio (ICER). The ICER therefore represents the additional expenditure associated with obtaining one additional unit of QEO through robotic-assisted rehabilitation compared with conventional rehabilitation. Because QEOs were estimated from functional outcomes rather than directly measured with preference-based instruments, the ICERs should not be interpreted as conventional cost-per-QALY estimates. They should instead be viewed as exploratory estimates of the potential economic value of robotic-assisted rehabilitation under the model assumptions, providing information for implementation and policy discussions rather than definitive evidence of cost-effectiveness in Indonesia.

### 2.5 Implementation and technology adoption framework

A rehabilitation perspective was primarily adopted, while also considering factors that may influence the implementation of robotic-assisted therapy (RAT) in clinical practice. The analysis incorporated viewpoints from healthcare providers, research institutions, professional organizations, industry stakeholders, and academic experts. This approach was used to examine not only the clinical value of RAT but also factors that may affect its adoption and long-term sustainability in Indonesia, including workforce readiness, infrastructure availability, economic feasibility, and stakeholder acceptance.

To examine these factors systematically, implementation evidence was organized into thematic domains reflecting key dimensions of technology adoption in rehabilitation settings, including clinical practice integration, health system readiness, workforce capacity, infrastructure constraints, and socio-cultural acceptance. These domains guided the narrative synthesis of implementation-related findings presented in the Results section.

Consumer acceptance of robotic rehabilitation technologies was considered as an additional dimension of feasibility, particularly in relation to adoption readiness and long-term sustainability within routine clinical practice.

## 3 Results

### 3.1 . Evidence synthesis and clinical effectiveness of robotic-assisted therapy

#### 3.1.1 Study characteristics and evidence distribution

A total of 22 systematic reviews and meta-analyses published between 2015 and 2025 were included in the qualitative evidence synthesis of robotic-assisted therapy (RAT) in stroke rehabilitation. Study characteristics and descriptive findings were manually extracted from each publication and organised through consensus-based categorisation among the reviewers. The synthesis focused on four predefined domains: rehabilitation intervention characteristics, biofeedback integration strategies, reported clinical outcomes, and the comparative effectiveness of RAT relative to conventional rehabilitation. Detailed characteristics of the included studies are summarised in Supplementary Tables S1–S2.

Robotic systems were designed to address specific rehabilitation goals. Upper-limb devices primarily targeted shoulder, wrist, and hand function, whereas lower-limb systems focused on gait-related impairments, including overground exoskeletons and treadmill-based multi-joint platforms. RAT enables high-intensity, task-specific, and repetitive training, particularly beneficial during early recovery phases. Several studies included participants across multiple stroke phases rather than focusing on a single recovery stage. Therefore, stroke-stage classifications were manually mapped and categorised based on the participant characteristics and descriptions reported in each study, using reviewer consensus to determine the most appropriate classification. Most studies were conducted predominantly in subacute stroke populations (*n* = 14), followed by chronic stroke populations (*n* = 12), while only a limited number focused on acute stroke rehabilitation (*n* = 3). Additionally, several studies did not clearly specify the stroke stage or included heterogeneous populations spanning multiple recovery phases (*n* = 8) (Figure 2).

**Figure 2:**
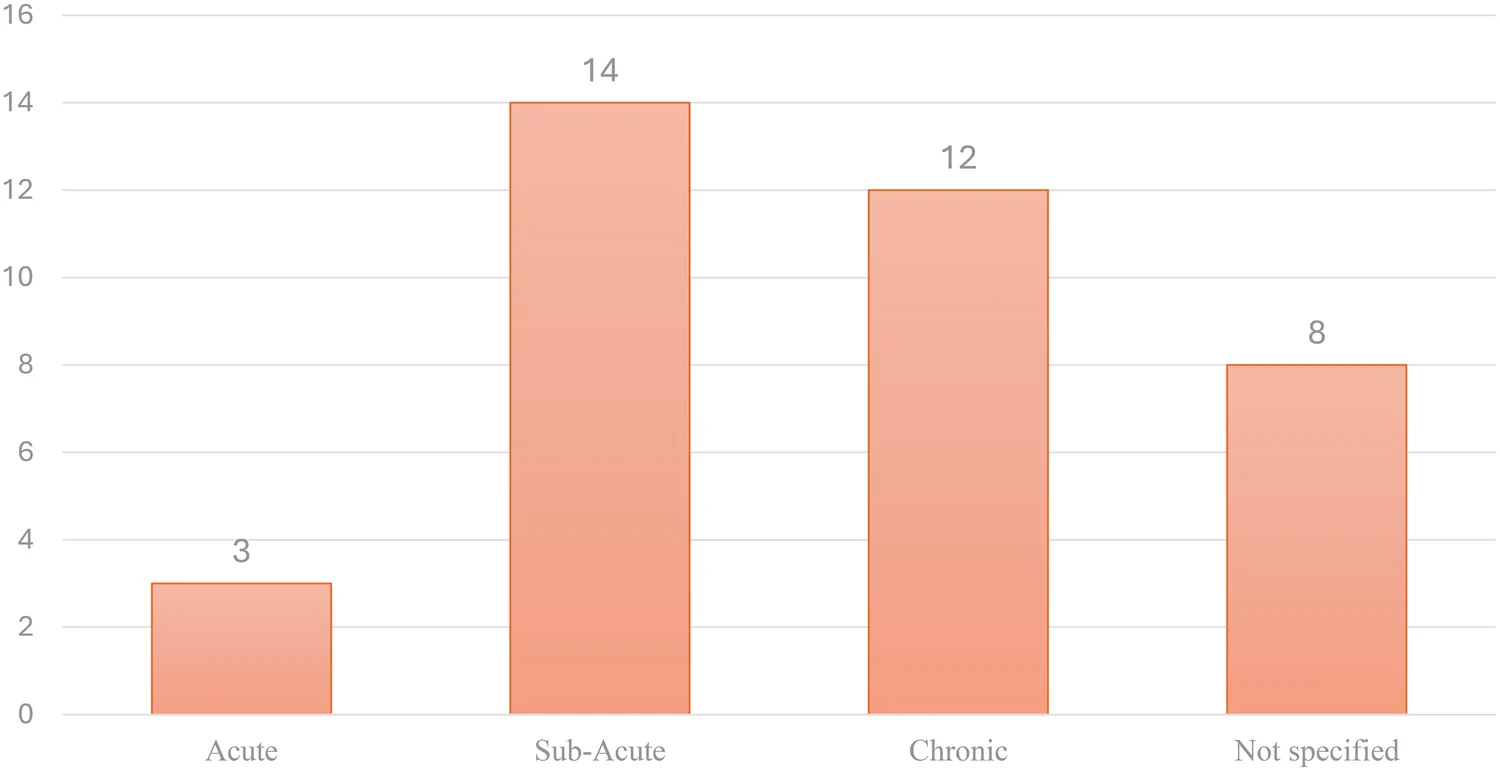
Distribution of stroke stages in 22 systematics reviews and meta analysis of robotic rehabilitation studies (2015–2025).

In terms of functional domains, most studies focused on lower-limb function (*n* = 7), followed by upper-limb rehabilitation (*n* = 6). Fewer studies addressed combined impairments (*n* = 2) or multidimensional outcomes such as balance, walking speed, or cardiovascular limitations (each *n* = 1) (Figure 3). Overall, the evidence base shows a strong emphasis on subacute rehabilitation and gait recovery.

**Figure 3:**
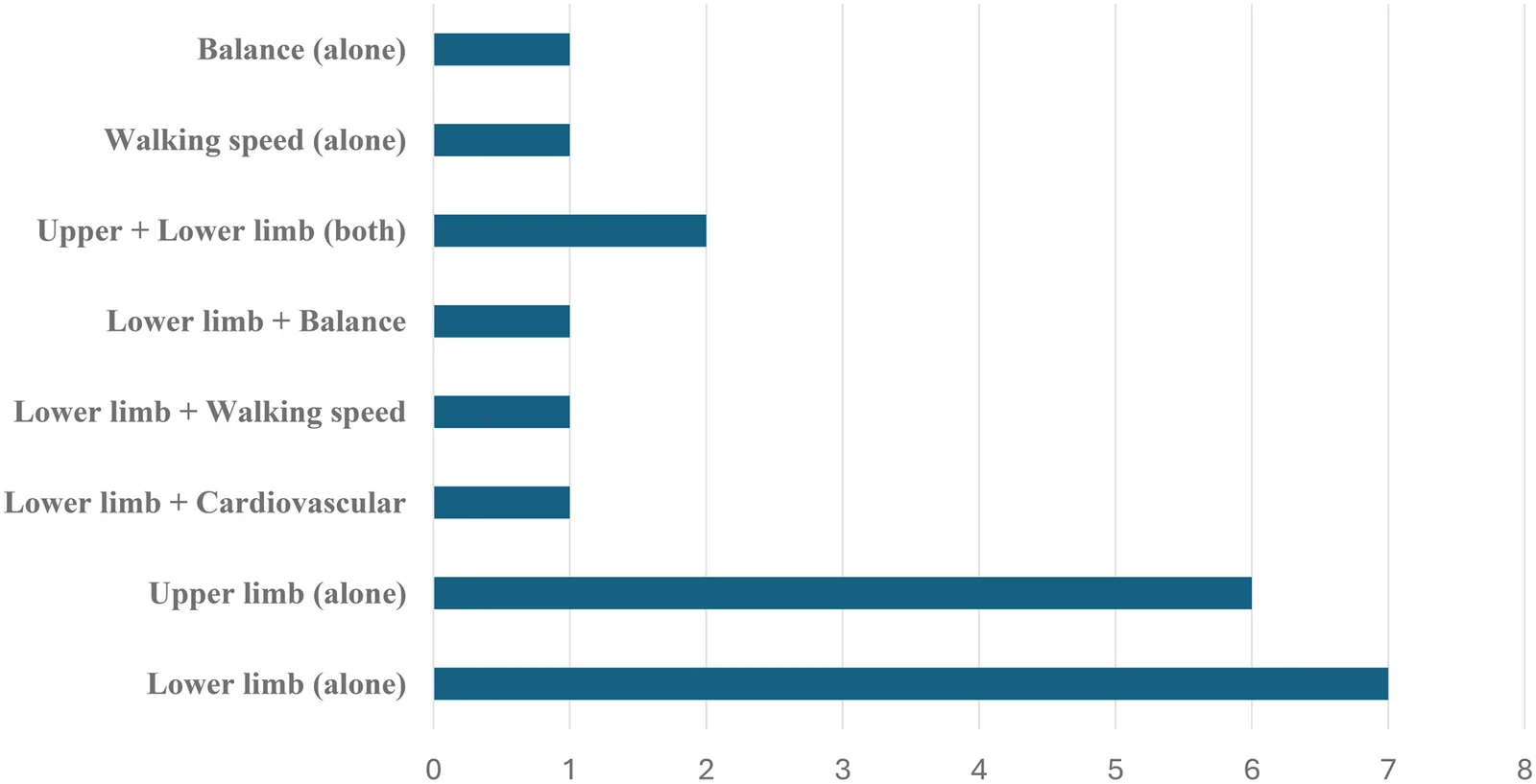
Classification of post-stroke conditions by functional impairment domain.

#### 3.1.2 Clinical outcomes and intervention characteristics

Across studies, a wide range of outcome measures were reported (Table 1). Upper-extremity outcomes included Fugl-Meyer Assessment (FMA-UE) and Action Research Arm Test (ARAT), while lower-extremity outcomes commonly involved gait speed, 6-Minute Walk Test (6MWT), Timed Up and Go (TUG), and Berg Balance Scale (BBS). General functional measures such as Functional Independence Measure (FIM), Barthel Index (BI), and SF-36 were also frequently used.

**Table 1:** Classification of outcome measures by affected limb (upper vs. lower extremity).

Category	Example measures
Upper extremity (UE)	FMA-UE, ARAT, BBT, WMFT, MAL, FM-UL, UL-capacity, MAS (upper limb)
Lower extremity (LE)	FMA-LE, Gait speed, 6MWT, 10MWT, FAC, BBS, TUG, RMI, Step length, Cadence
General/functional	FIM, BI, MBI, SIS, SF-36, EQ-5D, ADL, Motor recovery, Strength, VO_2_peak

Intervention protocols were descriptively classified according to reported rehabilitation dose and intensity characteristics (Table 2). Because several studies included heterogeneous or overlapping intervention designs, session frequencies and treatment intensities were manually curated to provide a rough comparative estimation across studies. Lower-limb robotic training generally involved sessions lasting 20–60 min, conducted 2–5 times per week over intervention periods of 2–8 weeks, corresponding to an estimated range of approximately 10–50 total sessions. Commonly reported devices included robotic gait trainers, body-weight–supported treadmill systems, exoskeletons, and virtual reality (VR)-enhanced rehabilitation platforms.

**Table 2:** Characteristics of rehabilitation programs using robotic assistive technologies.

Lower extremity/gait-based robotic training				
Treatment type	Session duration	Frequency	Total sessions/duration	Notes
Robot-assisted gait Training (RAGT)	30–60 min	3–5×/week	12–24 sessions (4–8 weeks)	Often combined with physiotherapy
Body-weight supported treadmill training (BWS-TT)	20–45 min	3–5×/week	12–20 sessions (3–6 weeks)	Used for gait and balance
Treadmill training (TT)	20–40 min	3–5×/week	12–24 sessions	Standard comparison group
Virtual reality (VR) gait training	30–45 min	2–5×/week	10–24 sessions	Often integrated with feedback
Overground walking training (OWT)	20–40 min	3–5×/week	12–20 sessions	With or without robotic aid
Exoskeleton-assisted gait training/powered exoskeleton (PAGT)	30–60 min	3–5×/week	10–40 sessions (≈2–8 weeks)	Devices: Ekso GT, HAL, GEMS, etc.
Lower limb robotic rehabilitation (LLRR)	30–60 min	3–5×/week	∼10–40 sessions	Lokomat, Walkbot, GaitMotus
Exosuit-assisted walking	20–40 min	2–5×/week	10–20 sessions (≈2–6 weeks)	Powered hip/ankle support
Lokomat® training (treadmill-based)	45–120 min	2–5×/week	8–60 sessions	Adjustable speed/guidance
Combined RAGT + physiotherapy	30–60 min	2–5×/week	12–36 sessions (≈3–12 weeks)	Gait, balance, ADL focus
Upper extremity/arm-based robotic training				
Treatment type	Session duration	Frequency	Total sessions/duration	Notes
Robot-assisted upper limb training (RT)	30–120 min	5×/week	9–40 sessions	Devices: Armeo, MIT-MANUS, REAplan, Gloreha
Upper-limb exoskeleton or end-effector therapy	20–60 min	2–6×/week	4–54 sessions (2–9 weeks)	Motor relearning, functional activities
Robot-assisted upper limb + physiotherapy	30–60 min	3–5×/week	12–30 sessions (4–8 weeks)	Focus on motor recovery and ADL
Robot-assisted motor training (upper or lower)	30–60 min	1–5×/week	2–45 sessions (2–9 weeks)	Hospital/rehab setting
Robot-assisted training with cognitive or respiratory component	30–60 min	3–5×/week	12–36 sessions	Combined neurorehabilitation
Mixed/multimodal or hybrid robotic interventions				
Treatment type	Session duration	Frequency	Total sessions/duration	Notes
Robotic + conventional therapy (balance, gait, ADL)	30–60 min	3–5×/week	10–40 sessions (2–8 weeks)	Integrated rehabilitation
VR/feedback/biofeedback-based systems	15–40 min	3–5×/week	6–36 sessions	Motor + cognitive feedback
Therapeutic/assistive Multisession designs	30–60 min	1–5×/week	5–31 sessions (avg ≈ 13)	Varies by study design
Variable-dose robotic training (RCTs)	30–60 min	3–5×/week	12–36 sessions	Duration 4–44 h total
Robot-assisted training with conventional OT/PT	∼45 min	4×/week	20–30 sessions	Integrated inpatient rehab

Upper-limb interventions demonstrated greater variability, ranging from 20 to 120 min per session and 1–6 sessions per week, resulting in an estimated total of 9–54 sessions. Frequently reported devices included Armeo, MIT-MANUS, and end-effector robotic systems designed to support motor learning, functional retraining, and activities of daily living (ADL)-focused rehabilitation. Several studies also described mixed or hybrid rehabilitation approaches integrating robotics with conventional therapy and/or VR-based interventions. Overall, this variability reflects differences in patient needs, rehabilitation goals, and institutional resources, highlighting the absence of standardized RAT protocols across clinical settings.

#### 3.1.3 Comparative effectiveness of RAT

Among the 22 included studies, 12 reported statistically significant improvements favouring robotic-assisted therapy (RAT) over conventional rehabilitation approaches, whereas two studies reported no significant benefit of RAT compared with standard treatment (Table 3). The remaining studies generally reported comparable or mixed findings depending on the rehabilitation domain, intervention protocol, or outcome measure evaluated. Effect estimates, including standardized mean differences (SMDs) and mean differences (MDs), were extracted where available.

**Table 3:** Comparative effects of robotic-assisted therapy (RAT) vs. conventional therapy in stroke rehabilitation.

Study	Robotic-assisted therapy (RAT)	Conventional therapy comparator	Main outcomes	Key findings/effect size	Overall conclusion	Reference no.
De Iaco et al.	Upper-limb robotic therapy	Conventional stroke rehabilitation/usual care	Upper-limb recovery, muscle power, ADL	FM-UL: MD = 2.23; Muscle power: SMD = 0.39; ADL: SMD = 0.28	Robotic UL therapy provides modest motor benefits	(45)
Loro et al.	Robot-assisted gait training (RAGT)	Physiotherapy, balance and gait training	Balance, gait, mobility	BBS: SMD = 0.47; TUG: MD = −4.05 s; FAC: SMD = 0.43	RAGT improves balance and walking function	(46)
Lyu et al.	RAGT, VR gait training, BWS treadmill training	Usual care/sham/no exercise	Dynamic balance	Dynamic balance improved (SMD = 0.25–0.41)	Robotic/VR gait interventions benefit balance recovery	(47)
Yang et al.	Robotic exoskeleton gait training	Conventional physiotherapy/rehabilitation	Gait speed, balance, motor recovery	FMA-LE: MD = 1.15; Gait velocity: MD = 0.09 m/s; BBS: MD = 2.34	Exoskeleton training enhances gait and mobility	(48)
Su et al.	Upper-limb robot-assisted rehabilitation	Conventional rehabilitation	Upper-limb function, ADL	FMA-UE: WMD = 5.27; MBI: WMD = 9.55	Robotic UL training improves arm recovery and daily activity	(49)
Hsu et al.	Wearable exoskeleton-assisted gait training	Conventional gait training	Walking speed, endurance, balance	Walking speed: MD = 0.13 m/s; Balance: SMD = 0.30; 6MWT: MD = 29.22 m	Wearable exoskeletons improve ambulatory performance	(50)
Wu et al.	Lower-limb robotic rehabilitation	Conventional physiotherapy/balance training	Cardiovascular fitness	VO_2_peak: SMD = 0.71	Robotic gait training may improve cardiovascular capacity	(51)
Hu et al.	Robot-assisted gait training	Conventional physiotherapy	Motor recovery, balance, walking independence	FMA-LE: MD = 5.48; FAC: MD = 0.57; BBS: MD = 4.48	RAGT effective in subacute stroke rehabilitation	(52)
Calafiore et al.	Lokomat/exoskeleton gait training	Conventional rehabilitation	Walking independence, gait function	No significant difference compared with conventional rehabilitation (NS)	Evidence remains inconclusive	(53)
Yang et al.	Upper-limb robotic therapy	Conventional physiotherapy/occupational therapy	Motor recovery, ADL	FMA-UE: SMD = 0.69; MBI: SMD = 0.95	Robotic UL therapy supports functional recovery	(54)
Chen et al.	Robot-assisted gait training	Conventional gait/treadmill training	Endurance, gait symmetry	6MWT: MD = 25.14 m; FAC: SMD = 0.32	Robotic gait training improves gait quality	(55)
Chien et al.	Upper-limb robotic therapy	Conventional therapy/usual care	Functional independence, motor control	Most pooled outcomes were non-significant (NS)	Limited evidence for benefit over standard therapy	(56)
Amirbekova et al.	Robotic motor rehabilitation	Conventional rehabilitation/physiotherapy	Overall motor recovery	Overall pooled effect: SMD = 0.59; stronger effects in acute/subacute stroke	Robotic rehabilitation more effective in earlier stroke phases	(57)
Wu et al.	Lokomat gait training	Conventional physical therapy	Balance, gait	BBS: MD = 2.71; gait speed and FIM were NS	Lokomat mainly benefits balance rather than gait speed	(58)

Overall, robotic-assisted therapy (RAT) demonstrated beneficial effects on motor recovery, gait performance, and balance compared with conventional rehabilitation in stroke patients. Significant improvements were reported in upper- and lower-limb motor function, including FMA-UE/FM-UL (MD up to 5.27) and FMA-LE (MD up to 5.48). Several studies also showed enhanced gait performance and balance, with improved walking speed (MD up to 0.13 m/s), Berg Balance Scale scores (MD up to 4.48), and walking endurance in the 6-Minute Walk Test (MD up to 29.22 m). However, some studies reported non-significant differences between RAT and conventional physiotherapy alone.

Despite these promising findings, the magnitude of benefit varied across studies, and several investigations reported non-significant outcomes when compared with conventional physiotherapy alone. For instance, Calafiore et al. and Chien et al. found no significant potentially beneficial of robotic interventions for several functional outcomes. Interpretation of the evidence is further limited by heterogeneity in robotic devices, treatment intensity, patient characteristics, and outcome measures.

Comparator interventions were also inconsistently described, often referred to broadly as “conventional rehabilitation,” “usual care,” or “dose-matched physiotherapy” without standardized treatment protocols. This inconsistency reduces the ability to determine equivalence between robotic and non-robotic therapies. Furthermore, many studies included relatively small sample sizes, moderate methodological heterogeneity, and limited long-term follow-up, which may reduce the generalizability and long-term applicability of the findings.

#### 3.1.4 Role of biofeedback in robotic rehabilitation

Biofeedback serves as an essential complement to robotic assisted therapy, transforming internal motor processes into observable signals that facilitate motor learning and real time error correction (21). Among the reviewed studies, visual (29%) and kinematic feedback (27%) were the most commonly used modalities, often delivered via robotic interfaces or virtual environments. Other modalities included proprioceptive feedback (11%), and haptic feedback (8%). Meanwhile, auditory feedback (about 9%) and electromyographic (EMG) feedback (nearly 4%) contribute additional layers of motor control, engagement, and user responsiveness (Figure 4).

**Figure 4:**
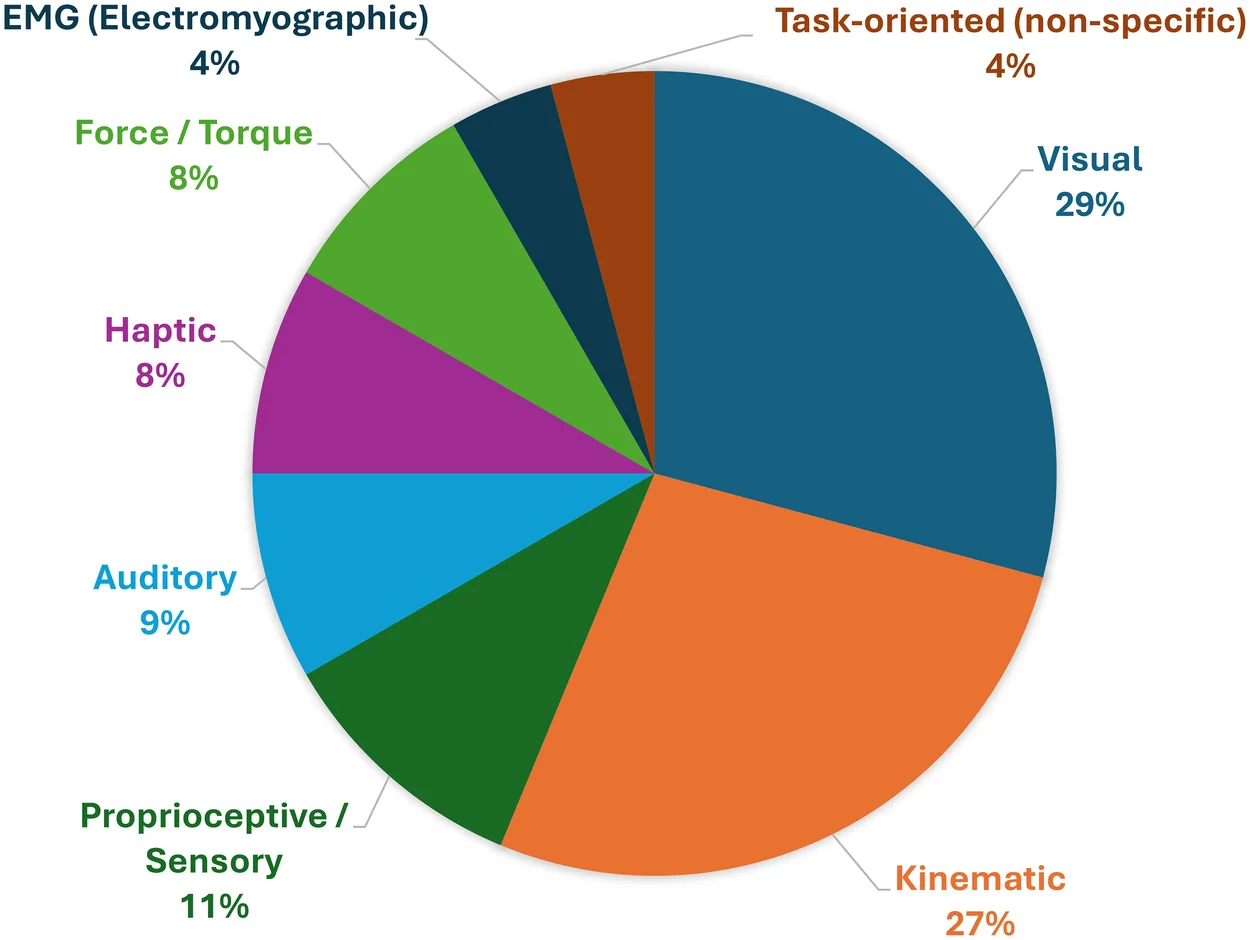
Distribution of biofeedback types used in robotic rehabilitation studies.

These feedback mechanisms can increase patient engagement, promote appropriate muscle activation, and help patients repeatedly practice task-specific movements, which may support neuroplastic changes (22, 23). However, the effectiveness of feedback depends on how it is delivered. Factors such as timing, frequency, and mode of feedback should be carefully adjusted to prevent cognitive overload and maintain patient motivation during training (24). Therefore, feedback strategies should be tailored to individual patient needs to improve motor learning, encourage long-term retention, and support sustained functional recovery.

### 3.2 . Model-based economy feasibility of robotic-assisted therapy in Indonesia

The economic feasibility of robotic-assisted therapy (RAT) in Indonesia is influenced by multiple cost drivers, including capital investment, regulatory requirements, and reimbursement limitations. RAT involves substantial upfront costs for device acquisition, alongside ongoing expenses for maintenance and consumables, which together increase the overall cost of intervention delivery from a healthcare system perspective.

From a regulatory standpoint, the adoption of RAT requires compliance with national medical device approval and importation procedures governed by Indonesian health authorities, which may add administrative and operational costs prior to clinical implementation (Permenkes No. 62/2017) (20). These requirements, combined with procurement and distribution processes, can contribute indirectly to increased intervention costs.

Reimbursement constraints further limit adoption Coverage for rehabilitation services under the Indonesian National Health Insurance system (BPJS Kesehatan/INA-CBGs), as regulated by the Ministry of Health (17), is implemented through a diagnosis-related group (case-based bundled payment) system and does not specify reimbursement for robotic-assisted rehabilitation technologies. Empirical data from BPJS Kesehatan claim samples for patients with cerebrovascular diseases (ICD-10 I60–I69) indicate substantial variability in reported reimbursement values across cases, from approximately Rp 340,523 to Rp 207,675,700 per patient per visit (≈USD 20–12,529) (25). This variation reflects heterogeneity in clinical severity, care pathways, and case grouping within the INA-CBG system rather than a technology-specific tariff. Consequently, no standardized reimbursement category currently exists for robotic-assisted therapy, which may create uncertainty in cost recovery and reduce institutional incentives for adoption.

To evaluate economic feasibility, a model-based economic analysis was conducted using parameters adapted from Shankar et al. (16) (Supplementary File S1). The model incorporated intervention costs, transition probabilities, and utility values to estimate QALY-equivalent outcomes (QEOs) from a healthcare system perspective. QEOs were derived using a ratio-based value-transfer approach that scaled a published reference QALY estimate according to the magnitude of functional improvement reported in the included rehabilitation studies. The incremental cost-effectiveness ratio (ICER) was subsequently calculated as the incremental cost per QEO and compared with willingness-to-pay (WTP) thresholds derived from Indonesia's gross domestic product (GDP) per capita.

Across the included studies, simulated ICERs for robotic-assisted therapy (RAT) varied according to intervention intensity and functional outcome domain, with most estimates remaining below the Indonesian willingness-to-pay (WTP) threshold based on gross domestic product (GDP) per capita (<US$5,030 per QEO). At low-to-moderate intervention intensities (8–24 sessions), ICER values consistently remained below the threshold across both upper- and lower-limb rehabilitation outcomes, suggesting potential cost-effectiveness under the study assumptions. Higher-intensity intervention schedules were associated with progressively increased ICER estimates, although several scenarios remained within the modelled cost-effectiveness range.

Regional analyses of the standardized 24-session robotic-assisted therapy (RAT) intervention demonstrated substantial variability in economic feasibility across Indonesian provinces. Provinces with higher gross regional domestic product (GRDP) per capita, including DKI Jakarta, Kalimantan Timur, and Kalimantan Utara, consistently met the willingness-to-pay (WTP) thresholds across most therapeutic scenarios. ICER estimates for upper-limb functional recovery (FM-UL/FMA-UE) ranged from US$236.23 to US$752.31 per QEO, whereas gait- and walking-related outcomes demonstrated comparatively higher ICER values ranging from US$558.86 to US$4,890 per QEO. Most provinces remained below their respective regional WTP thresholds, particularly for upper-limb rehabilitation domains. In contrast, provinces with lower economic indicators demonstrated reduced affordability for gait- and walking-related outcomes, highlighting persistent regional disparities in access to advanced rehabilitation technologies (Supplementary File S1). Overall, these findings suggest that RAT could be a potentially cost-effective option under the scenario-based model and policy assumptions applied in this study, particularly at low-to-moderate rehabilitation intensities within the Indonesian stroke rehabilitation setting.

### 3.3 . Implementation challenges in the Indonesian healthcare system

#### 3.3.1 Technical and infrastructure barriers

The implementation of robotic-assisted therapy (RAT) in Indonesia is primarily concentrated in Class A and B hospitals, which possess better infrastructure, funding capacity, and access to specialized personnel, including physiatrists, therapists, and biomedical engineers. These classifications are based on national hospital regulatory standards that define criteria such as human resources, medical facilities, equipment, and bed capacity, as stipulated in the Hospital Law (Law No. 44/2009) (18), the Minister of Health Regulation on Hospital Classification and Licensing (17), and Government Regulation on Hospital Administration (19). These institutions, often affiliated with universities or research centers, are therefore more capable of adopting and sustaining advanced rehabilitation technologies. In contrast, Class C and D hospitals face substantial limitations in infrastructure, workforce availability, and financial resources, restricting equitable access to RAT.

Additional technical barriers include the limited availability of robotic devices, which are predominantly imported and regulated through national medical device approval systems. Medical devices must comply with importation, registration, and utilization procedures regulated by the Ministry of Health (Kemenkes) through the Directorate General of Pharmaceutical Services and Medical Devices (Ditjen Farmalkes), including risk classification and conformity with international standards (CE, FDA, ISO).

As RAT systems are predominantly imported, additional requirements include a Local Authorized Representative (LAR) with Medical Device Distributor License (IDAK) and Good Distribution Practice for Medical Devices (CDAKB), as well as a Marketing Authorization Number (NIE). Compliance with local content requirements (TKDN) under the Ministry of Industry (Kemenperin) further affects procurement feasibility. Additional collaboration with the National Research and Innovation Agency (BRIN) and National Accreditation Committee (Komite Akreditasi Nasional, KAN may be required for testing and conformity assessment, while post-market surveillance is mandatory after deployment.

These interrelated regulatory, technical, and institutional requirements are summarized within a broader health system framework illustrating the external ecosystem, internal hospital capacity, and feedback mechanisms that shape the implementation and sustainability of RAT in Indonesia (Figure 5).

**Figure 5:**
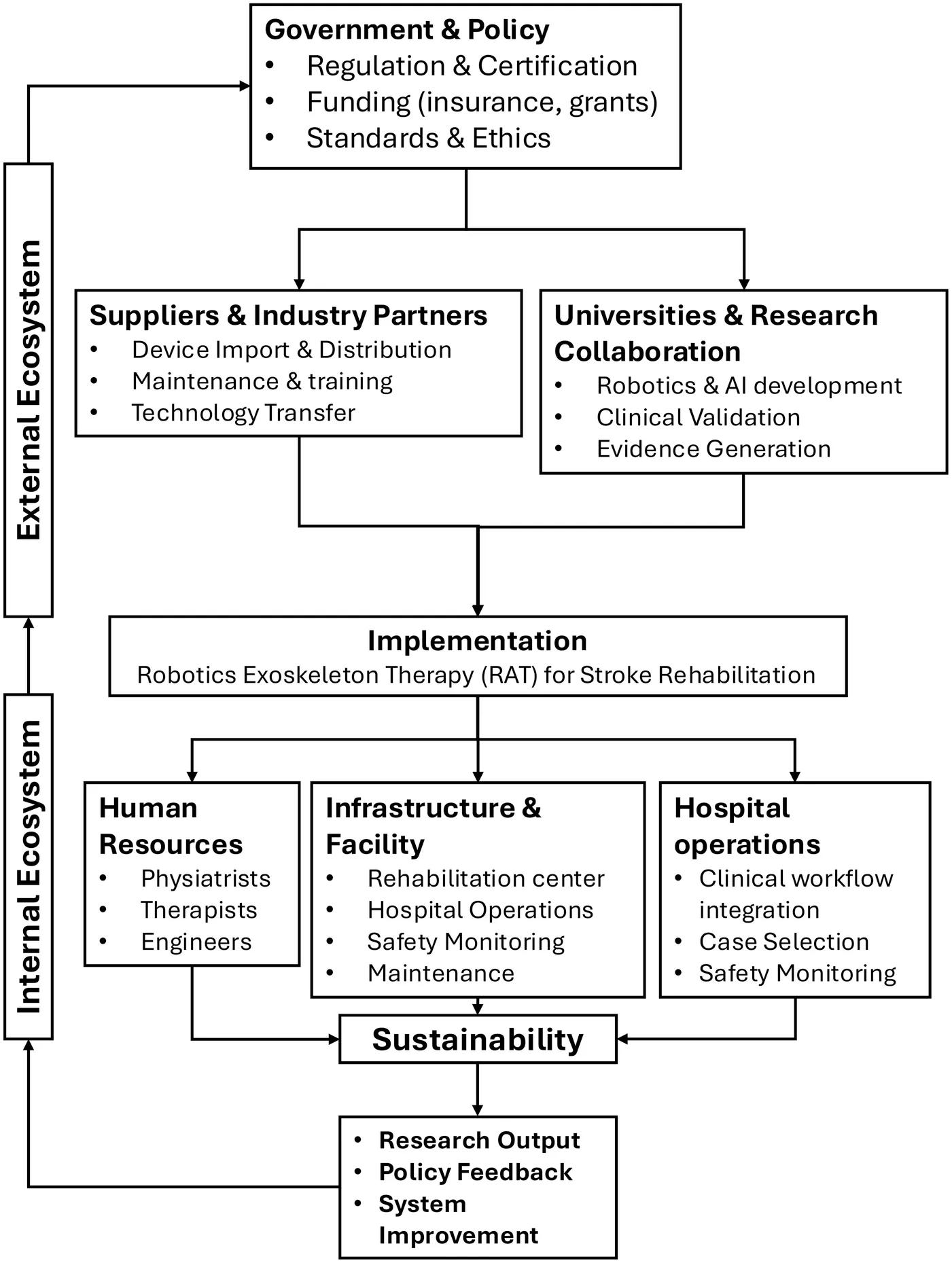
Implementation framework for robotic-assisted rehabilitation within the Indonesian health system.

#### 3.3.2 Workforce, training, and education barriers

From a workforce perspective, successful RAT implementation requires trained rehabilitation professionals (26), yet structured training opportunities remain limited in Indonesia, resulting in a persistent skills gap. Addressing this gap requires the development of multidisciplinary training pipelines, including early-stage strategies such as pilot programs and short-term industry-linked training, alongside long-term integration of RAT competencies into higher education curricula aligned with national qualification frameworks (SN-Dikti and KKNI) (27).

Accreditation bodies (e.g., LAM-PTKes, LAM Teknik, LAM INFOKOM, and BAN-PT) play a critical role in ensuring training quality and standardization, while professional associations and the Ministry of Health (Kemenkes) support competency definition, clinical guidelines, and certification. Supplementary Table S3 outlines the conceptual training program and learning modules for RAT in stroke rehabilitation, proposed by the authors to support potential integration into clinical training pathway.

Sustainable workforce development further depends on coordination between academic institutions, healthcare providers, and industry partners to ensure a continuous supply of qualified personnel and the long-term integration of RAT into clinical practice (Figure 6).

**Figure 6:**
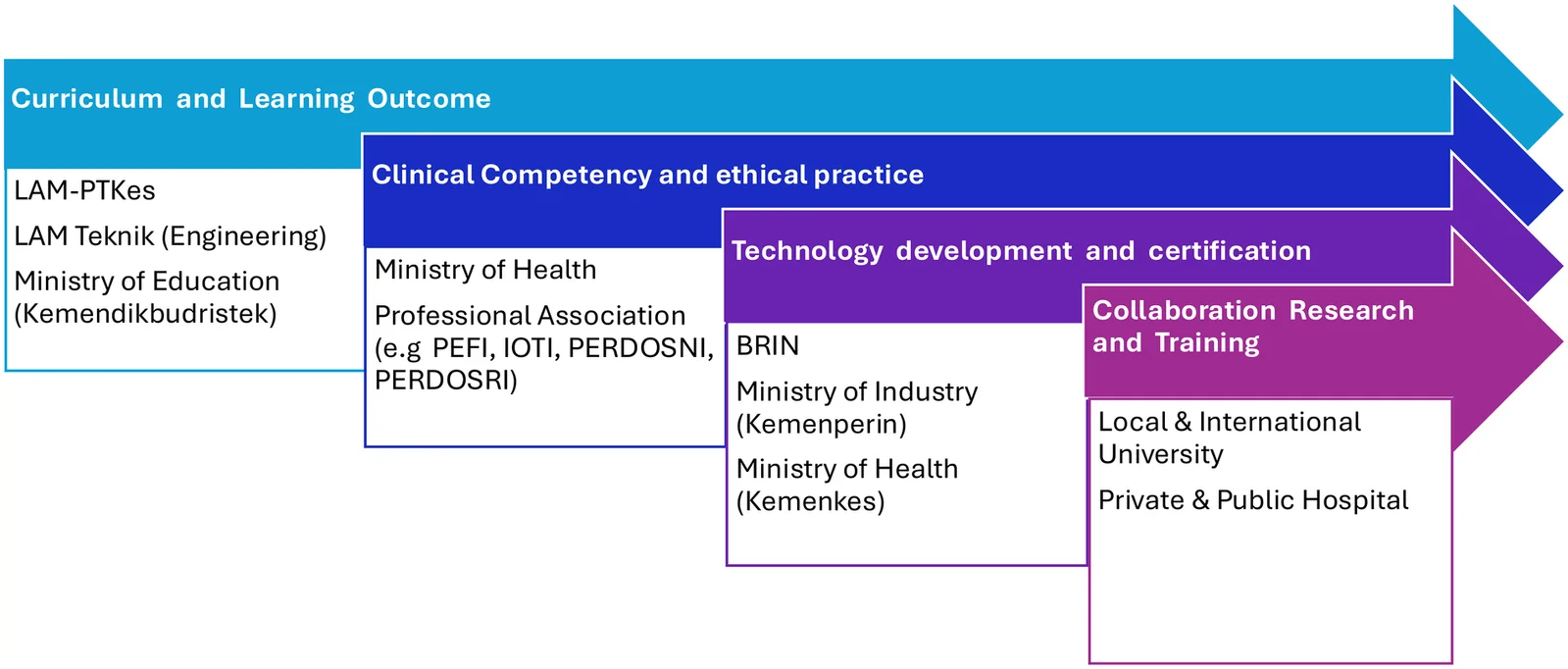
Stakeholder framework for education, clinical practice, and research in robotics assistive technologies.

#### 3.3.3 Cultural and adoption factors

Cultural perceptions and contextual factors are key influences on the adoption of robotic-assisted therapy (RAT) in Indonesia. From the patient perspective, robotic rehabilitation may initially be perceived as complex, intimidating, or unnecessary compared to conventional physiotherapy, which potentially limiting acceptance and engagement (28). Prior study highlighted perceived usefulness, ease of use, social influence, and digital health literacy as strong predictors of willingness to adopt digital and robotic health interventions (29, 30). However, empirical evidence on these factors within the Indonesian context remains limited, highlighting an important gap for future research. To address this gap, a context-specific evaluation framework has been proposed (Supplementary Table S4) to assess patient acceptance of RAT in stroke rehabilitation.

Clinician acceptance is another key factor influencing implementation success. Some practitioners view robotic technologies as innovative tools, whereas others express concerns regarding economic feasibility, workflow disruption, and clinical utility. Perceived usefulness, effort expectancy, and facilitating conditions, including training availability, technical support, and organizational support, are commonly associated with clinician adoption (31, 32). In practice, workflow compatibility and time efficiency are central to successful integration.

Professional organizations and institutional leadership play an important role in supporting adoption by ensuring that robotic technologies complement rather than replace conventional therapy, while also providing guidance, training, and support. Additionally, factors such as professional autonomy, clinical judgment, and trust in system reliability further influence clinician willingness to adopt these technologies (33, 34). Overall, integration of RAT in Indonesia requires consideration of both patient and clinician perspectives, along with cultural attitudes and system readiness.

## 4 Discussion

Across the included reviews, robotic-assisted therapy (RAT) consistently demonstrated benefits for motor recovery after stroke. However, successful implementation was influenced by several factors, including equipment costs, therapist training, patient acceptance, and healthcare infrastructure. These factors may limit wider adoption in Indonesia, where rehabilitation resources remain unevenly distributed and differences in healthcare capacity and access may lead to varying feasibility of RAT implementation across settings. The consistency of motor recovery benefits across studies suggests that the therapeutic potential of RAT is likely to be transferable to Indonesian patients, whereas the feasibility of achieving these benefits in routine practice is less certain because it depends on the availability of appropriate infrastructure, trained personnel, financial resources, and technical support. Thus, clinical effectiveness may be broadly generalizable, but its translation into routine practice remains context dependent. This review extends the existing clinical evidence by highlighting that RAT adoption cannot be determined by clinical effectiveness alone, but also requires consideration of economic feasibility, implementation barriers, and the healthcare context in which the technology is introduced.

Robotic-assisted therapy appears to be most clinically relevant during the subacute phase, when recovery is strongly driven by repeated task practice and neuroplastic adaptation. Robotic-assisted therapy (RAT) is generally linked to improvements in motor function, gait performance, and activities of daily living in stroke rehabilitation; however, these effects are not uniform across patient groups, with outcomes strongly influenced by stroke severity, targeted function, and intervention design. Improvements in specific outcomes such as muscle strength and spasticity appear less consistent across studies, suggesting that certain impairments may be less responsive to robotic interventions than broader functional outcomes (35–37). These findings indicate that treatment response is unlikely to be uniform across stroke populations. As a result, evidence from one rehabilitation setting or patient group should not automatically be generalized to others.

One practical implication is that RAT may be most useful when integrated into existing rehabilitation programs rather than used as a stand-alone intervention. Conventional physiotherapy still provides important clinical flexibility and individualized decision-making that robotic systems cannot fully replace. However, conventional rehabilitation is not a uniform intervention and may involve different treatment approaches depending on the targeted function, patient characteristics, and clinical setting. Therefore, the relative effectiveness of RAT should be interpreted in relation to the specific conventional treatment used as the comparator rather than assuming that conventional therapy represents a standardized intervention. Combining both approaches may therefore allow patients to benefit from intensive repetitive training while retaining therapist-guided adaptation settings (38–40). This approach may also help reduce treatment costs in settings where extensive robotic use is not feasible.

Several methodological issues make interpretation of the current evidence difficult. Differences in training intensity and device functionality likely affect direct comparison across studies. Many studies also characterized by short follow-up periods, making it difficult to determine long-term effectiveness and cost-efficiency. In addition, stroke subtypes were often poorly reported, reducing the ability to identify which patient groups are most likely to benefit from RAT, as highlighted by Dierick et al. (2017) (41). Furthermore, Cohen's kappa was not calculated to formally quantify inter-reviewer agreement during study screening and data extraction. These limitations should be considered when interpreting the current evidence base.

In Indonesia, the high acquisition and maintenance costs of robotic systems, together with complex regulatory requirements and limited reimbursement coverage, appear to constrain scalability within a resource-limited healthcare system. Consistent with the economic modelling findings reported by Shankar et al. (16), our analysis suggests that not all robotic interventions may represent economically feasible options under the model assumptions used in this study when compared with conventional therapy. Acceptable incremental cost-effectiveness ratios (ICERs) were largely observed in major urban centres such as Jakarta and in regions with relatively high average incomes. Expanding access to lower-resource settings may therefore depend on additional investment, broader reimbursement support, and the availability of locally generated economic data. Although long-term maintenance, spare-parts availability, and technical support are recognized as important considerations for sustainable RAT implementation, these aspects were not further analysed within the proposed scope of this review, particularly given the limited Indonesia-specific evidence available.

Nevertheless, these findings should be interpreted cautiously, as the analysis is based on modelled assumptions rather than real-world cost and reimbursement data from Indonesian rehabilitation settings. Because this structured narrative synthesis did not formally assess the methodological quality of the underlying systematic reviews or independently pool their estimates, the reported clinical effect estimates remain dependent on the quality and limitations of those reviews. In particular, uncertainty remains regarding key cost drivers such as robotic device pricing, institutional rehabilitation costs, and differences in healthcare infrastructure across regions, all of which are likely to influence ICER estimates but could not be fully captured in this study. In addition, reliance on GDP per capita–based willingness-to-pay thresholds may not adequately reflect actual healthcare access, particularly in lower-resource settings where individual affordability can vary substantially.

A further limitation relates to the economic modelling approach itself. The QALY-equivalent outcomes estimates were not derived from validated utility instruments such as EQ-5D or SF-6D but were instead approximated using value-transfer methods based on functional outcomes reported in the included literature (16). As a result, the ICER estimates should be viewed as preliminary, providing directional rather than definitive cost-utility information. Moreover, this study did not conduct full health technology assessment modelling, probabilistic sensitivity analysis, or long-term state-transition analysis due to limited availability of cost, utility, and utilization data in the Indonesian context. Consequently, the findings are intended to inform early-stage discussion on integration and policy development rather than support direct reimbursement decisions.

Workforce readiness is likely to be a major challenge for the sustainable adoption of RAT in Indonesia. International studies have consistently shown that clinicians often perceive limited training opportunities and a lack of hands-on experience as key barriers to implementation (15, 42, 43). These challenges may be particularly relevant in Indonesia, where access to specialized rehabilitation services remains uneven across regions. As a result, efforts to expand RAT should focus not only on acquiring technology but also on developing a workforce that is adequately trained to use and integrate these systems into clinical practice.

Even when infrastructure and workforce capacity are sufficient, the technology acceptance is another factor that may influence whether RAT becomes part of routine clinical practice. Patients generally report positive experiences with robotic rehabilitation, particularly when therapy is engaging and perceived as beneficial (44). These findings are derived primarily from studies conducted outside Indonesia, and no Indonesia-specific empirical study of RAT acceptance among stroke survivors or rehabilitation professionals was identified in this review. Therefore, findings regarding patient and clinician attitudes should be considered as contextual evidence rather than direct evidence of Indonesian perceptions. Clinical attitudes appear more mixed due to concern about workflow disruption, additional training requirements, and uncertainty regarding clinical value are commonly reported. Addressing these concerns may require not only technical training but also greater exposure to clinical evidence and practical implementation experience. Support from professional organizations and hospital leadership may help build confidence and encourage adoption over time.

In conclusion, current evidence supports the potential role of robotic-assisted therapy in stroke rehabilitation, particularly for improving motor recovery and functional performance. However, clinical effectiveness alone is unlikely to determine adoption in Indonesia. Infrastructure availability, workforce capacity, reimbursement mechanisms, and user acceptance are equally important considerations. Given these challenges, a gradual implementation strategy beginning with pilot programs and tertiary-care settings may be more realistic than large-scale deployment.

This may include pilot implementation programs in high-volume Class A academic or tertiary hospitals, with DKI Jakarta and other regions considered where the specified clinical-volume, workforce, infrastructure, and monitoring criteria are met. Initial implementation could prioritize settings with sufficient clinical capacity and technical support, followed by conditional expansion to selected Class B referral hospitals once acceptable clinical outcomes, utilization, safety, economic feasibility, and technology acceptance have been demonstrated. At later stages, hybrid rehabilitation models, workforce training, context-specific policy development, and appropriate reimbursement mechanisms may further support wider adoption of robotic-assisted rehabilitation in Indonesia. Future studies should focus on generating local clinical, economic, and implementation data, including evidence on technology acceptance, to better inform policy decisions regarding rehabilitation robotics in Indonesia.
