# Supplementary material for: Implementing robotic-assisted therapy in stroke rehabilitation: a structured narrative review of evidence and technology adoption barriers in Indonesia
Source: Front Rehabil Sci. 2026 Oct 8;7:1942904. doi: 10.3389/fresc.2026.1942904 (PMC13687834; doi:10.3389/fresc.2026.1942904)
Supplement: Supplementary file 1 [file Supplementaryfile1.docx]

**Supplementary File S1**

**Economic Modelling and Cost-Effectiveness Scenario Analysis Using QALY-Equivalent Outcomes (QEOs)**

In this part, we are adapted the economic evaluation framework proposed by Shankar et al. (2025) to develop a pragmatic cost-effectiveness model for robotic-assisted therapy (RAT) in stroke rehabilitation within the Indonesian healthcare context. Because direct economic evaluations of robotic-assisted therapy are not currently available in Indonesia, an exploratory modelling approach was developed using published rehabilitation evidence, reimbursement data, and national economic indicators. This modelling exercise was designed to assess whether robotic-assisted therapy could achieve cost-effectiveness ratios that fall within commonly applied Indonesian willingness-to-pay thresholds when evaluated using published effectiveness estimates and local reimbursement data.

Because the included studies reported different clinical outcomes, a scaling approach was applied to express treatment effects within a common economic framework. This method retained the relative differences across studies but did not assume that the outcomes were directly comparable. The purpose of this approach was not to generate absolute patient-level utility values, but to estimate the relative health benefits needed to determine whether robotic-assisted rehabilitation could remain economically viable under realistic reimbursement and affordability conditions in Indonesia.

However, this proxy utility scaling method has several limitations. Functional improvements may not correspond proportionally to changes in health-related quality of life. In addition, the rehabilitation outcome measures included in the pooled analysis capture different dimensions of health and function and therefore cannot be considered equivalent to utility-based outcomes. Consequently, deriving QALYs from pooled effect sizes may add uncertainty to the estimates and affect the precision of the economic evaluation. The adapted framework integrates two complementary dimensions of economic evaluation:

1. **Economic justification**, assessed using Incremental Cost-Effectiveness Ratio (ICER) relative to willingness-to-pay (WTP) thresholds; and
2. **Practical affordability**, assessed by comparing estimated intervention costs with regional healthcare reimbursement capacity and regional economic indicators.

This dual-layer framework was intended to provide a regionally grounded estimation of whether robotic-assisted rehabilitation could achieve clinically meaningful outcomes while remaining financially sustainable for large-scale implementation.

**Data Collection and Modelling**

The economic simulation incorporated evidence from systematic reviews and meta-analyses of robotic-assisted stroke rehabilitation, regional economic statistics, and healthcare reimbursement data. Data Integration and Analytical Framework show in figure 1:

a. **Robotic Rehabilitation Cost Assumptions**, Fixed robotic rehabilitation costs were adapted from Shankar et al. (2025), which estimated a six-month robotic rehabilitation cost of USD 2,560.59 per patient based on:
- robotic device acquisition cost of USD 209,642;
- annual maintenance cost of USD 10,360;
- and an estimated device lifespan of eight years.

These values were used as baseline reference costs for economic modelling.

b. **Rehabilitation Program Characteristics,** Characteristics of rehabilitation programs are summarized in **Table 1**. Among the included systematic reviews and meta-analyses, 6 of 22 studies reported sufficient statistical information for economic modelling. For these studies, mean differences between robot-assisted therapy (RAT) and conventional therapy were available and appears to be adequate for inclusion in the economic model. Based on the reported intervention characteristics across studies, the duration of robotic rehabilitation was assumed to be approximately eight weeks (two months), reflecting the most commonly reported treatment period in the included literature.

To enable comparison across studies, upper-limb and lower-limb outcomes were grouped into two broad functional domains: motor recovery and gait performance. This approach allowed outcomes measured using different instruments to be incorporated into a common modelling framework. Measures such as BBS, TUG, FAC, 6MWT, and ARAT were retained for clinical interpretation but were excluded from QALY equivalent estimate scaling because their units and measurement constructs were not directly comparable.

c. **Healthcare Reimbursement Assumptions,** National Health Insurance reimbursement data from Indonesia (BPJS Kesehatan) were used as the minimum reimbursement benchmark. Based on Santoso, Adisasmito, and Hasibuan (2024), the estimated minimum reimbursement value was USD 20.55 per rehabilitation visit (IDR 340,523; exchange rate IDR 16,570/USD).
d. **Regional Economic Indicators and WTP Thresholds.** We assume, based on World Bank data, that Indonesia’s GDP per capita is approximately USD 5,000–6,000 per year, while regional GDP may vary (Badan Pusat Statistik, 2025). This information is used as a macroeconomic benchmark reflecting population-level opportunity cost proxies in cost-effectiveness analysis. In this context, the willingness-to-pay (WTP) threshold is applied as a policy decision rule to represent how much health gain was worth paying for under Indonesian health system and the robotic-assisted rehabilitation (RAT) context, rather than reflecting individual financial capacity or actual household expenditure behaviour.

Figure.1 Model Framework and Analytical workflow. The analytical workflow consisted of three stages: (1) data input from regional GDP, QALY estimates, and insurance coverage; (2cost-effectiveness calculation using adapted Shankar framework and QEO scaling; and (3) ICER-to-WTP comparison for affordability evaluation.

1. **Derivation of QALY-Equivalent Outcomes (QEOs) Using Mean-Difference Scaling**

To estimate QALY-equivalent outcomes (QEOs), a value-transfer approach was applied because direct utility measurements (e.g., EQ-5D) were unavailable in the included studies. This study uses a value-transfer and scenario simulation framework rather than a primary trial-based economic evaluation. Mean differences were used as relative indicators of treatment effect and served as scaling factors within the scenario model. No direct mapping from functional outcomes to utility scores was performed. QALY estimation was anchored on external economic evidence from a previous cost-effectiveness analysis of robotic exoskeleton therapy versus conventional physiotherapy in stroke rehabilitation in Singapore by Shankar et al., which reported utility-based QALY gains ranging from **0.13 to 0.22 over 6 months. These published QALY values were used solely as anchor values for deriving QEOs.** A midpoint value of 0.175 was used as the base-case reference for model calibration under utility range scenarios range 0.13 to 0.22.

QEOs for each included study were estimated by scaling a reference value using the ratio of observed mean differences (MD) in functional outcomes relative to a reference MD within the same clinical domain (e.g., upper or lower limb rehabilitation). This approach was used to harmonize heterogeneous clinical outcomes into a common economic metric while preserving relative treatment effects. The estimates were applied as scenario-based approximations and compared with published economic evaluations rather than treated as direct utility measurements. A value-transfer approach was used in which functional MD served as scaling factors to adapt external utility-based estimates to the Indonesian setting, where direct utility data for robotic-assisted therapy were not available, assuming proportional transferability of treatment effects to health utility while acknowledging uncertainty in cross-setting extrapolation.

$${QEO}_{i}=0.175 \times\frac{{MD}_{i}}{{MD}_{ref}}$$

Where :

${QEO}_{i}$	= Projected QEO of study (i) using relative treatment effect (MD ratios)

${MD}_{i}$	= Mean Difference of relative effectiveness of study (i)

${MD}_{ref}$	= Mean Difference from the reference from the same domain of Upper Extremity or Lower extremity treatment effect

Under utility range scenario analysis:

$${QEO}_{i}^{low}=0.13 \times\frac{{MD}_{i}}{{MD}_{ref}}$$

$${QEO}_{i}^{high}=0.22 \times\frac{{MD}_{i}}{{MD}_{ref}}$$

Table 1. Domain-specific QALY-equivalent outcomes (QEO) using ratio-based scaling of functional mean differences

| **Study No** | **Stroke Phase** | **Domain** | **Selected Outcome** | **MD Used** | **QEO Study-Specific (Base)** | **QEO Low** | **QEO High** | **Notes / Interpretation** |
| --- | --- | --- | --- | --- | --- | --- | --- | --- |
| 2 | Acute/Subacute | Upper Limb (UL) | FM-UL | 2.23 | 0.175 | 0.13 | 0.22 | Reference UL anchor; moderate functional recovery with proportional utility gain |
| 7 | Subacute-Chronic | Lower Limb (LL) | Gait velocity | 0.09 m/s | 0.121 | 0.09 | 0.152 | Improvement in gait performance; lower than LL reference effect |
| 8 | Chronic | Upper Limb (UL) | FMA-UE | 5.27 | 0.414 | 0.307 | 0.52 | Strong upper-limb motor recovery; highest projected UL utility gain |
| 10 | Subacute-Chronic | Lower Limb (LL) | Walking speed | 0.13 m/s | 0.175 | 0.13 | 0.22 | Reference LL anchor; clinically meaningful gait-speed improvement |
| 14 | Subacute | Lower Limb (LL) | Walking speed | 0.10 m/s | 0.135 | 0.1 | 0.169 | Moderate gait-speed improvement with projected functional mobility benefit |
| 22 | Mixed | Lower Limb (LL) | Gait speed | 0.02 m/s | 0.027 | 0.02 | 0.034 | Minimal clinical effect; limited projected utility benefit |

Note : QEO = QALY-equivalent outcome derived using ratio-based scaling of reference utility values; not directly measured QALYs.

2. **Calculation per-visit price with Indonesia affordability constraint**

Next, the economic model adopted the six-month benefit horizon reported by Shankar et al. (2025), which estimated a total six-month robotic rehabilitation cost of USD 2,560.59 per patient in Singapore. Because the original study did not specify the exact number of rehabilitation sessions delivered during the intervention period, the present study applied a standardized utilization assumption to estimate per-session costs for comparative economic modelling.

Based on the rehabilitation characteristics reported across the included robotic-assisted therapy studies (main manuscript **Table 2**), robotic rehabilitation programs commonly involved multiple weekly treatment sessions over approximately 6 months. Therefore, an estimate utilization scenario of four sessions per week over 26 weeks was applied, corresponding to an estimated total of 104 rehabilitation sessions per patient, then:

$$C_{per visit}= \frac{C_{total 6 mo}}{total visits}= \frac{2.560,59}{104}=24,62 USD/visit$$

This assumption was used solely to standardize cost distribution across treatment sessions and does not represent an exact utilization value from the original study from Shankar et al. (2025). This assumption enabled a transparent and reproducible estimate of approximate per-session robotic rehabilitation costs under Indonesian conditions.

In the main-manuscript review of Table-2, the treatment session corresponds to approximately **8 to 54 total visits** with consideration of 24 as ideal treatment, then:

Minimal	:8 visits	→ total cost ≈ 8 × 24.62 ≈ **197 USD**

Ideal/median	: 24 visits	→ total cost ≈ 24 × 24.62 ≈ **591 USD**

Maximal	: 54 visits	→ total cost ≈ 54 × 24.62 ≈ **1.329 USD**

The **Incremental Cost** function is then defined as:

$$Incremental Cost (\boldsymbol{\Delta C)}=C_{robotic}- C_{conventional therapy}$$

Since Indonesia currently has no reported cost data for Robotic Assistive Therapy and relies solely on conventional therapy for stroke patients, we used the national health insurance reimbursement rate reported by **Santoso, Adisasmito, and Hasibuan (2024)**, which is approximately **Rp 340.523,-** (USD 20.55) per visit. With the same visit count then we can calculate:

Table 2. Incremental Cost Calculations Based on Visit Counts

| **Visit count** | **Robotic total Cost/ visit**  **(Shankar-adjusted)** | **Conventional**  **(Covered by Indonesia**  **National Health Insurance)** | **ΔC (USD)** |
| --- | --- | --- | --- |
| 8 | 197 USD | 8 × 20,55 = 164,40 USD | 32,60 |
| 24 | 591 USD | 24 × 20,55 = 493,20 USD | 97,80 |
| 54 | 1,329 USD | 54 × 20,55 = 1.109,70 USD | 219,30 |

Based on Incremental Cost (**ΔC)** then we can calculate Incremental Cost-Effectiveness Ratio (ICER) by combining the result of QEO estimates

$$ICER= \frac{Incremental Cost}{QALY gain per year}$$

GDP per capita was used as reference threshold because it reflects the broader economic capacity of the healthcare system and is commonly applied in exploratory cost-effectiveness analyses conducted in resource-limited settings. According to the **IMF World Economic Outlook (April 2025)**, Indonesia’s GDP per capita is approximately **USD 5,030**, which roughly represents the average economic capacity of the country, that is, the amount of income available per person within the economy. GDP-based thresholds were used as practical reference points rather than direct estimates of opportunity cost. These thresholds were chosen because Indonesia does not currently have an established willingness-to-pay threshold for robotic-assisted rehabilitation.

Then to ensures the intervention provide additional health benefits at a cost that public health considers worth paying, then

$$if ICER<WTP threshold\to Cost-Effective$$

$$if ICER>WTP threshold\to Not Cost-Effective$$

For example, ICER of effectivity robotic assistive therapy technology for Motor Recovery in upper limb for acute/subacute stroke patients for 8 visit:

$$ICER=\frac{Incremental Cost \left( \boldsymbol{\Delta C} \right)\boldsymbol{for 8 visit count}}{\boldsymbol{QEO gain (Upper Limb FM-UL-acute subacute stroke condition)}}$$

**ICER** = 32.60 / 0.175 ≈ **186.28 USD per QEO**

Then :

$$ICER \left( 8 visit for \boldsymbol{Upper Limb FM-UL-acute subacute stroke condition} \right)<WTP threshold (GDP per capita Indonesia)$$

$$186.28 USD<5030 USD$$

Because cost-effectiveness analysis primarily evaluates the efficiency of healthcare resource allocation under limited health budgets, willingness-to-pay (WTP) thresholds were applied in this study as policy-oriented decision rules to determine whether the health benefits generated by robotic-assisted rehabilitation (RAT) could be considered economically justifiable in Indonesian healthcare setting. Therefore, the WTP threshold in the present analysis does not represent individual financial capacity or actual household spending behaviour but rather reflects the estimated level of health gain that may reasonably be supported by the healthcare system relative to available economic resources.

Within this framework (**Table 3**), the calculated incremental cost-effectiveness ratio (ICER) of USD 186.28 per QALY-equivalent outcomes (QEO) over the assumed six-month therapeutic benefit period remained substantially below even the lower Indonesian willingness-to-pay (WTP) threshold range. The QEO in this study represent scaled short-term functional utility gains associated with robotic-assisted rehabilitation (RAT) compared with conventional therapy, based on the six-month benefit horizon adapted from Shankar et al. (2025), rather than yearly nor lifetime utility projections. Columns highlighted in grey indicate that the intervention may not be cost-effective for certain stroke conditions, outcome categories, or treatment frequencies under the selected regional economic thresholds (ICER> GDP per capita). Moreover, the QEO metric should be interpreted as a utility-based proxy outcome derived from published reference values and functional effect scaling rather than as a directly measured quality-adjusted life year.

Based on these findings, the 24-visit rehabilitation scenario was selected for subsequent economic modelling because it represented a balanced treatment duration capable of capturing most of the estimated therapeutic utility associated with robotic-assisted rehabilitation (RAT) across the included studies. This scenario may be the most clinically and economically representative for comparative implementation analysis.

The selected 24-visit ICER estimates were subsequently applied to regional economic modelling across Indonesian provinces using regional Gross Domestic Product (GDP) data and updated currency exchange values based on the most recent economic information available in May 2026. This method was selected to evaluate the potential affordability and economic feasibility of robotic-assisted rehabilitation under varying regional economic conditions within Indonesia.

Province-level modelling demonstrated substantial regional variation in the economic feasibility of robotic-assisted rehabilitation across Indonesia (**Table 4**). Under the 24-visit scenario, five of six rehabilitation profiles remained below provincial GDP-based thresholds in all provinces, whereas the mixed lower-limb gait-speed profile exceeded thresholds in several lower-income provinces. This suggests that affordability is sensitive to both treatment effectiveness and regional economic capacity.

Several limitations should be considered when interpreting the province-level cost-effectiveness analysis in this study. First, the use of regional GDP per capita as a proxy willingness-to-pay threshold may not fully reflect the actual economic capacity of individual populations within each province, particularly in regions with substantial income inequality and socioeconomic disparity. Consequently, provincial economic indicators may overestimate the practical affordability of robotic-assisted rehabilitation for certain communities and healthcare institutions.

Second, the present analysis did not account for differences in healthcare infrastructure availability, rehabilitation workforce distribution, technological readiness, or accessibility of specialized medical equipment across provinces. In many regions of Indonesia, advanced rehabilitation technologies and healthcare resources remain concentrated in major urban centers or provincial capitals such as DKI Jakarta, whereas rural and remote areas may have limited access to rehabilitation facilities capable of implementing robotic-assisted therapy.

Additionally, regional healthcare policies and medical technology distribution strategies may not necessarily align with economic equality across all provinces. The concentration of investment, infrastructure development, and high-cost medical technologies in economically developed cities may create disparities between estimated affordability and real-world implementation capacity. For these reasons, the province-level estimates should be viewed as broad economic scenarios rather than direct predictions of implementation success.

Table 3. National-Level QALY-Equivalent Utility Scaling and ICER Estimates for Robotic-Assisted Rehabilitation Across Different Visit Scenarios

| Study No | Condition | Domain | Selected Outcome | MD Used | QEO (Base) | QEO Low | QEO High | ICER for 8 visits (QALY) | ICER for 24 visits (QALY) | ICER for 54 visits (QALY) |
| --- | --- | --- | --- | --- | --- | --- | --- | --- | --- | --- |
| 2 | Acute/Subacute | Upper Limb (UL) | FM-UL | 2.23 | 0.175 | 0.13 | 0.22 | 186.29 (250.77 - 148.18) | 558.86 (752.31 - 444.55) | 1253.14 (1686.92 - 996.82) |
| 7 | Subacute-Chronic | Lower Limb (LL) | Gait velocity | 0.09 m/s | 0.121 | 0.09 | 0.152 | 269.42 (362.22 - 214.47) | 808.26 (1086.67 - 643.42) | 1812.40 (2436.67 - 1442.76) |
| 8 | Chronic | Upper Limb (UL) | FMA-UE | 5.27 | 0.414 | 0.307 | 0.52 | 78.74 (106.19 - 62.69) | 236.23 (318.57 - 188.08) | 529.71 (714.33 - 421.73) |
| 10 | Subacute-Chronic | Lower Limb (LL) | Walking speed | 0.13 m/s | 0.175 | 0.13 | 0.22 | 186.29 (250.77 - 148.18) | 558.86 (752.31 - 444.55) | 1253.14 (1686.92 - 996.82) |
| 14 | Subacute | Lower Limb (LL) | Walking speed | 0.10 m/s | 0.135 | 0.1 | 0.169 | 241.48 (326.00 - 192.90) | 724.44 (978.00 - 578.70) | 1624.44 (2193.00 - 1297.63) |
| 22 | Mixed | Lower Limb (LL) | Gait speed | 0.02 m/s | 0.027 | 0.02 | 0.034 | 1207.41 (1630.00 - 958.82) | 3622.22 (4890.00 - 2876.47) | 8122.22 (10965.00 - 6450.00) |

Note :

- UL = Upper Limb; LL = Lower Limb; FM-UL and FMA-UE both refer to the upper-extremity component of the Fugl–Meyer Assessment evaluating upper-limb motor recovery after stroke.
- Walking speed and gait speed represent lower-limb locomotor performance outcomes and are used interchangeably across included studies to assess ambulatory recovery after stroke

Table 4. Province-Level Cost-Effectiveness Feasibility of Robotic-Assisted Rehabilitation Using

GDP-Based Willingness-to-Pay Thresholds in Indonesia (1)

| Province | Per Capita Gross Regional Domestic Product | Approx USD (May 2026) 17680,90/USD | Stroke Phase | Acute/Subacute | Subacute-Chronic | Chronic | Subacute-Chronic | Subacute | Mixed |
| --- | --- | --- | --- | --- | --- | --- | --- | --- | --- |
|  |  |  | Domain | Upper Limb (UL) | Lower Limb (LL) | Upper Limb (UL) | Lower Limb (LL) | Lower Limb (LL) | Lower Limb (LL) |
|  |  |  | Outcome Category | FM-UL | Gait velocity | FMA-UE | Walking speed | Walking speed | Gait speed |
|  |  |  | Threshold: | 558.86  (752.31 - 444.55) | 808.26  (1086.67 - 643.42) | 236.23  (318.57 - 188.08) | 558.86  (752.31 - 444.55) | 724.44  (978.00 - 578.70) | 3622.22  (4890.00 - 2876.47) |
| Aceh | Rp45,770,000 | USD 2,589 |  | ✔ | ✔ | ✔ | ✔ | ✔ | ✘ |
| Sumatera Utara | Rp78,310,000 | USD 4,429 |  | ✔ | ✔ | ✔ | ✔ | ✔ | ✘ |
| Sumatera Barat | Rp59,549,000 | USD 3,368 |  | ✔ | ✔ | ✔ | ✔ | ✔ | ✘ |
| **Riau** | **Rp176,385,000** | **USD 9,976** |  | **✔** | **✔** | **✔** | **✔** | **✔** | **✔** |
| **Jambi** | **Rp92,786,000** | **USD 5,248** |  | **✔** | **✔** | **✔** | **✔** | **✔** | **✔** |
| Sumatera Selatan | Rp80,663,000 | USD 4,562 |  | ✔ | ✔ | ✔ | ✔ | ✔ | ✘ |
| Bengkulu | Rp52,305,000 | USD 2,958 |  | ✔ | ✔ | ✔ | ✔ | ✔ | ✘ |
| Lampung | Rp55,009,000 | USD 3,111 |  | ✔ | ✔ | ✔ | ✔ | ✔ | ✘ |
| Kepulauan Bangka Belitung | Rp75,323,000 | USD 4,260 |  | ✔ | ✔ | ✔ | ✔ | ✔ | ✘ |
| **Kepulauan Riau** | **Rp172,459,000** | **USD 9,754** |  | **✔** | **✔** | **✔** | **✔** | **✔** | **✔** |
| **DKI Jakarta** | **Rp367,687,000** | **USD 20,796** |  | **✔** | **✔** | **✔** | **✔** | **✔** | **✔** |
| Jawa Barat | Rp59,865,000 | USD 3,386 |  | ✔ | ✔ | ✔ | ✔ | ✔ | ✘ |
| Jawa Tengah | Rp50,824,000 | USD 2,875 |  | ✔ | ✔ | ✔ | ✔ | ✔ | ✘ |
| DI Yogyakarta | Rp55,039,000 | USD 3,113 |  | ✔ | ✔ | ✔ | ✔ | ✔ | ✘ |
| Jawa Timur | Rp80,856,000 | USD 4,573 |  | ✔ | ✔ | ✔ | ✔ | ✔ | ✘ |
| Banten | Rp74,673,000 | USD 4,223 |  | ✔ | ✔ | ✔ | ✔ | ✔ | ✘ |
| Bali | Rp72,658,000 | USD 4,109 |  | ✔ | ✔ | ✔ | ✔ | ✔ | ✘ |
| Nusa Tenggara Barat | Rp33,667,000 | USD 1,904 |  | ✔ | ✔ | ✔ | ✔ | ✔ | ✘ |
| Nusa Tenggara Timur | Rp25,837,000 | USD 1,461 |  | ✔ | ✔ | ✔ | ✔ | ✔ | ✘ |

Table 4. Province-Level Cost-Effectiveness Feasibility of Robotic-Assisted Rehabilitation Using

GDP-Based Willingness-to-Pay Thresholds in Indonesia (2)

| Province | Per Capita Gross Regional Domestic Product | Approx USD (May 2026) 17680,90/USD | Stroke Phase | Acute/Subacute | Subacute-Chronic | Chronic | Subacute-Chronic | Subacute | Mixed |
| --- | --- | --- | --- | --- | --- | --- | --- | --- | --- |
|  |  |  | Domain | Upper Limb (UL) | Lower Limb (LL) | Upper Limb (UL) | Lower Limb (LL) | Lower Limb (LL) | Lower Limb (LL) |
|  |  |  | Outcome Category | FM-UL | Gait velocity | FMA-UE | Walking speed | Walking speed | Gait speed |
|  |  |  | Threshold: | 558.86  (752.31 - 444.55) | 808.26  (1086.67 - 643.42) | 236.23  (318.57 - 188.08) | 558.86  (752.31 - 444.55) | 724.44  (978.00 - 578.70) | 3622.22  (4890.00 - 2876.47) |
| Kalimantan Barat | Rp56,984,000 | USD 3,223 |  | ✔ | ✔ | ✔ | ✔ | ✔ | ✘ |
| Kalimantan Tengah | Rp84,730,000 | USD 4,792 |  | ✔ | ✔ | ✔ | ✔ | ✔ | ✘ |
| Kalimantan Selatan | Rp70,577,000 | USD 3,992 |  | ✔ | ✔ | ✔ | ✔ | ✔ | ✘ |
| **Kalimantan Timur** | **Rp208,331,000** | **USD 11,783** |  | **✔** | **✔** | **✔** | **✔** | **✔** | **✔** |
| **Kalimantan Utara** | **Rp208,210,000** | **USD 11,776** |  | **✔** | **✔** | **✔** | **✔** | **✔** | **✔** |
| Sulawesi Utara | Rp75,235,000 | USD 4,255 |  | ✔ | ✔ | ✔ | ✔ | ✔ | ✘ |
| **Sulawesi Tengah** | **Rp131,643,000** | **USD 7,445** |  | **✔** | **✔** | **✔** | **✔** | **✔** | **✔** |
| Sulawesi Selatan | Rp78,748,000 | USD 4,454 |  | ✔ | ✔ | ✔ | ✔ | ✔ | ✘ |
| Sulawesi Tenggara | Rp72,031,000 | USD 4,074 |  | ✔ | ✔ | ✔ | ✔ | ✔ | ✘ |
| Gorontalo | Rp47,477,000 | USD 2,685 |  | ✔ | ✔ | ✔ | ✔ | ✔ | ✘ |
| Sulawesi Barat | Rp46,654,000 | USD 2,639 |  | ✔ | ✔ | ✔ | ✔ | ✔ | ✘ |
| Maluku | Rp33,652,000 | USD 1,903 |  | ✔ | ✔ | ✔ | ✔ | ✔ | ✘ |
| **Maluku Utara** | **Rp97,262,000** | **USD 5,501** |  | **✔** | **✔** | **✔** | **✔** | **✔** | **✔** |
| **Papua Barat** | **Rp139,213,000** | **USD 7,874** |  | **✔** | **✔** | **✔** | **✔** | **✔** | **✔** |
| Papua Barat Daya | Rp61,624,000 | USD 3,485 |  | ✔ | ✔ | ✔ | ✔ | ✔ | ✘ |
| Papua | Rp84,305,000 | USD 4,768 |  | ✔ | ✔ | ✔ | ✔ | ✔ | ✘ |
| Papua Selatan | Rp64,928,000 | USD 3,672 |  | ✔ | ✔ | ✔ | ✔ | ✔ | ✘ |
| **Papua Tengah** | **Rp107,566,000** | **USD 6,084** |  | **✔** | **✔** | **✔** | **✔** | **✔** | **✔** |
| Papua Pegunungan | Rp19,111,000 | USD 1,081 |  | ✔ | ✔ | ✔ | ✔ | ✔ | ✘ |

**References**

1) Shankar R, Tang N, Shafawati N, Phan P, Mukhopadhyay A, Chew E. Cost-effectiveness analysis of robotic exoskeleton versus conventional physiotherapy for stroke rehabilitation in Singapore from a health system perspective. BMJ Open. 2025 Jul 7;15(7):e095269. doi: 10.1136/bmjopen-2024-095269. PMID: 40623888; PMCID: PMC12273162.
2) Santoso M, Adisasmito WBB, Hasibuan SR. Cost of illness: Stroke patients with medical rehabilitation. *J Keterapian Fisik.* 2024;9(1). doi:10.37341/jkf.v9i1.422
3) International Monetary Fund. (2025). *World Economic Outlook: GDP per capita, current prices — Indonesia* [Data set]. IMF. <https://www.imf.org/external/datamapper/NGDPDPC%40WEO/IDN>
4) Badan Pusat Statistik. (2026, March 10). Produk domestik regional bruto per kapita atas dasar harga berlaku menurut provinsi (ribu rupiah). Retrieved May 15, 2026, from <https://www.bps.go.id/en/statistics-table/3/YWtoQlRVZzNiMU5qU1VOSlRFeFZiRTR4VDJOTVVUMDkjMw%3D%3D/produk-domestik-regional-bruto-per-kapita-atas-dasar-harga-berlaku-menurut-provinsi>
