# Supplementary material for: Implementing robotic-assisted therapy in stroke rehabilitation: a structured narrative review of evidence and technology adoption barriers in Indonesia
Source: Front Rehabil Sci. 2026 Oct 8;7:1942904. doi: 10.3389/fresc.2026.1942904 (PMC13687834; doi:10.3389/fresc.2026.1942904)
Supplement: Supplementary file 2 [file Table1.docx]

**Supplementary Table S1. Types of Robotic Devices for Stroke Rehabilitation and Their Therapeutic Goals**

| **No.** | **Author** | **Year** | **Study Design** | **Population** | **Condition** | **Intervention** | **General Outcome** | **Ref.** | |
| --- | --- | --- | --- | --- | --- | --- | --- | --- | --- |
| **1** | Jaya Shanker Tedla, Snehil Dixit, Kumar Gular, Mohammed Abohashrh | 2019 | Systematic Review & Meta-analysis | Subacute and chronic stroke survivors (>18 years) | Post-stroke gait impairment (subacute & chronic stroke) | Robotic-Assisted Gait Training (RAGT), sometimes combined with conventional physiotherapy (CPT) | RAGT showed similar or slightly better effects than conventional gait training (CGT) on gait speed and functional outcomes; subgroup: chronic stroke had slight positive effect | 1 |  |
| **2** | Luca De Iaco, Janne M. Veerbeek, Johannes C.F. Ket, Gert Kwakkel | 2024 | Systematic Review and Meta-analysis | 4,311 stroke patients with upper limb motor impairment | Post-stroke upper limb impairment | Upper Limb Robot Therapy (UL-RT) | Small but significant improvements in muscle synergy (FM-UL), muscle power, self-reported performance, and basic ADLs. No significant overall effects for UL-capacity (except exoskeletons). | 2 |  |
| **3** | Elizabeth C. Wonsetler, PT, DPT; Mark G. Bowden, PhD, PT | 2018 | Systematic review of clinical intervention studies | Adults (>18 yr) with clinically diagnosed stroke (mostly chronic; 3 studies sub-acute; 1 compared sub-acute & chronic) | Post-stroke gait impairment with reduced self-selected walking speed | Physical-therapy–based gait rehabilitation interventions (10 categories: aerobic, functional electrical stimulation, hippotherapy, motor dual-task, multidimensional rehab, robotics, sensory stimulation, strength/resistance, task-specific locomotor, visually-guided training) | Physical-therapy–based gait rehabilitation interventions (10 categories: aerobic, functional electrical stimulation, hippotherapy, motor dual-task, multidimensional rehab, robotics, sensory stimulation, strength/resistance, task-specific locomotor, visually-guided training) | 3 |  |
| **4** | Pinheiro, C.; Figueiredo, J.; Cerqueira, J.; Santos, C.P. | 2022 | Scoping Review | Post-stroke patients, median sample size = 17 (range varied) | Post-stroke gait disability | Robotic biofeedback systems (visual, auditory, haptic cues) during gait or related motor tasks | Improvement in gait parameters (step length, symmetry, walking speed), motor recovery, neuromuscular coordination | 4 |  |
| **5** | Loro, A.; Borg, M.B.; Battaglia, M.; Amico, A.P.; Antenucci, R.; Benanti, P.; Bertoni, M.; Bissolotti, L.; Boldrini, P.; Bonaiuti, D.; et al. | 2022 | Systematic Review & Meta-Analysis | Post-stroke patients (acute, subacute, chronic phases; various disability levels) | Stroke-related gait and balance impairment | Robot-Assisted Gait Training (RAGT) + Conventional Therapy vs. Conventional Therapy alone | Improvement in balance and functional mobility | 5 |  |
| **6** | Tianyi Lyu, Kang Yan, Jiaxuan Lyu, Xirui Zhao, Ruoshui Wang, Chaoyang Zhang, Meng Liu, Chao Xiong, Chengjiang Liu, Yulong Wei | 2023 | Systematic Review & Network Meta-Analysis | 2,328 adult stroke survivors (mean age 44.2–74.8 yrs; 56.6% male) | Post-stroke balance impairment | Six gait training types: treadmill (TT), body-weight-supported treadmill (BWS-TT), virtual reality gait training (VR), robotic-assisted gait training (RA-GT), overground walking training (OWT), conventional gait training (CGT) | Balance outcomes (dynamic steady-state, static steady-state, proactive, balance test batteries) | 6 |  |
| **7** | Juncong Yang, Yongxin Zhu, Haojie Li, Kun Wang, Dan Li, Qi Qi | 2024 | Systematic Review & Meta-analysis | 1,166 stroke patients (mean age ~34–72 years across studies) | Stroke survivors with lower limb motor dysfunction | Lower Limb Robotic Exoskeleton Training (LRET), often combined with conventional physiotherapy (CPT) | Improvements in lower limb motor control, gait parameters, walking independence, balance, activities of daily living, and participation | 7 |  |
| **8** | Tingting Su, Mengting Wang, Zhouyang Chen, Liang Feng. | 2024 | Systematic review & meta-analysis | 573 stroke patients across 18 RCTs (mean age ≈ 52 in RT group, 56 in CR group). Recovery phases included both subacute (≤6 months) and chronic (>6 months). | Post-stroke upper limb motor impairment / hemiplegia. | Upper-limb robot-assisted training (RT) — various devices; trials compared RT versus conventional rehabilitation (CR). Some trials used robots as the main modality, others as adjunct to CR per trial design. | RT superior to CR for upper-limb motor impairment, functional arm tests, activities of daily living, and reduced muscle tone. | 8 |  |
| **9** | Yang Yang, Mingzhe Xie, Hongyu Wang, Yufei Zhang, Hongmei Liu, and Jie Jia | 2023 | Systematic Review | Chronic stroke survivors (10 RCTs, total N ≈ 313) | Chronic stroke with gait impairment | Exoskeleton robot-assisted training (RAT) | RAT improved walking independence, balance, and endurance compared with conventional therapy; however, effects on gait speed were inconsistent and evidence quality ranged from low to moderate. | 9 |  |
| **10** | Ting-Hsuan Hsu, Chi-Lin Tsai, Ju-Yang Chi, Chih-Yang Hsu, Yen-Nung Lin | 2022 | Systematic Review and Meta-analysis | 13 studies, 492 post-stroke participants (mixed ambulatory and non-ambulatory, mean age 50–71 years, stroke onset 1–75 months) | Post-stroke gait impairment | Powered overground wearable exoskeleton robot-assisted gait training (PAGT) | PAGT was superior to dose-matched conventional gait training (CGT) in walking speed, balance at intervention end, and mobility & endurance at follow-up. No significant difference in ADL or motor function. | 10 |  |
| **11** | Yu Wu, Jian Liu, Moneruzzaman Md, Jun Zhao, Shicai Wu | 2024 | Systematic Review and Meta-analysis | 5 RCTs, 179 post-stroke patients (mean age ≈ 60.8 years; 62% male) | Post-stroke, lower limb motor and cardiovascular impairment | Lower limb robotic rehabilitation (LLRR) combined with conventional therapy | LLRR significantly improved peak oxygen uptake (VO₂peak). No significant effects on resting heart rate (HRrest), peak systolic BP (SBPpeak), or peak diastolic BP (DBPpeak). | 11 |  |
| **12** | Ya‑Chi Chuang, Yu‑Lin Tsai, Tony Tung‑Liang Lin, Liang‑Jun Ou‑Yang, Yu‑Chun Lee, Yuan‑Yang Cheng,  Chuan‑Ching Liu and Chun‑Sheng Hsu | 2023 | Systematic Review | Stroke patients with gait impairments; small sample sizes (individual studies: n = 1–20; total < 100 across all studies) | Post-stroke hemiparesis with reduced ambulation ability | Soft robotic exosuit (wearable exosuits assisting hip and/or ankle movement during walking) | Improved walking speed, endurance, and symmetry during gait; assistance reduced energy cost of walking | 12 |  |
| **13** | Cora Carrillo, Devyn Tilley, Kaitlyn Horn, Michelle Gonzalez, Cassidy Coffman,  Claudia Hilton, and Karthik Mani | 2023 | Systematic Review | Post-stroke patients with upper extremity motor impairment; mixed subacute and chronic phases; adults | Stroke-related upper extremity dysfunction | Robotic-assisted therapy (RAT) for upper limb rehabilitation, sometimes combined with conventional therapy | Improvements in motor recovery, functional ability, and ADLs, especially when RAT combined with conventional therapy | 13 |  |
| **14** | Miao‑miao Hu, Shan Wang, Cai‑qin Wu, Kun‑peng Li, Zhao‑hui Geng, Guo‑hui Xu and Lu Dong | 2024 | Systematic Review & Meta-analysis | 457 subacute stroke patients (≤6 months post-stroke) | Post-stroke gait and lower extremity functional impairment | Robot-Assisted Gait Training (RAGT), sometimes combined with Conventional Physiotherapy (CPT) | RAGT significantly improved lower extremity motor function, gait independence, and activities of daily living compared with CPT alone | 14 |  |
| **15** | Campagnini S., Liuzzi P., Mannini A., Riener R., Carrozza M. C. | 2022 | Systematic review | Post-stroke adults (subacute & chronic phases; pooled N across included studies = 247 post-stroke patients) | Stroke-related gait impairment | Robot-assisted gait training / exoskeletons using different control strategies (position, force/impedance, assist-as-needed, tunnel, bang-bang, hybrid, EMG/EEG inputs) | Reported improvements in kinematic, spatiotemporal and kinetic gait parameters in many studies; no clear controller “winner” due to heterogeneity | 15 |  |
| **16** | Dario Calafiore, Francesco Negrini, Nicola Tottoli, Francesco Ferraro, Ozden Ozyemisci-Taskiran, Alessandro de Sire | 2022 | Systematic review with meta-analysis | 576 subacute stroke survivors (≤6 months post-stroke) across 14 RCTs | Subacute stroke with gait impairment | Robot-assisted gait training (RAGT) using lower-limb robotic exoskeletons (Lokomat, HAL, Ekso, Walkbot) | Gait recovery: Functional Ambulation Category (FAC), gait speed, 6-minute walk test (6MWT), 10-meter walk test (10MWT), Timed Up & Go (TUG), stride/stance parameters | 16 |  |
| **17** | Xinwei Yang, Xiubo Shi, Xiali Xue, Zhongyi Deng | 2023 | Systematic review and meta-analysis | 1,019 stroke patients in total (sum of all participants across the included RCTs: 514 in robot-assisted training groups and 505 in control/conventional therapy groups). Adults with ischemic or hemorrhagic stroke, across acute, subacute, and chronic phases depending on individual trial | Post-stroke upper-limb motor impairment | Robot-assisted upper-limb training (end-effector or exoskeleton devices) | Upper-limb functional recovery: Fugl-Meyer Assessment–Upper Extremity (FMA-UE), Action Research Arm Test (ARAT), Box and Block Test (BBT), Wolf Motor Function Test (WMFT), Barthel Index (BI/ADL), Modified Ashworth Scale (MAS for spasticity) | 17 |  |
| **18** | Chen S., Zhang W., Wang D., Chen Z. | 2024 | Systematic review & meta-analysis | 1167 stroke patients (607 intervention, 560 control) | Post-stroke walking dysfunction / lower limb motor impairment | Robot-Assisted Gait Training (RAGT), including Exoskeletons and End-Effector devices, treadmill-based (T-RAGT) and overground (O-RAGT) | RAGT improved functional ambulation and endurance, particularly: – Better step length (affected side) – Improved temporal symmetry – Increased 6MWT distance (walking endurance) – Higher Functional Ambulation Category scores (independence) But no significant differences in walking speed, cadence, stride length, spatial symmetry, or joint mobility compared with conventional rehab. Benefits more pronounced in acute stroke and when combined with conventional therapy. | 18 |  |
| **19** | Wai-tong Chien, Yuen-yu Chong, Man-kei Tse, Cheuk-woon Chien, Ho-yu Cheng | 2020 | Systematic Review & Meta-analysis | 11 RCTs, n=493, Adults (18–65 yrs), subacute (<6 months post-stroke) | Subacute stroke with upper-limb impairment | Robot-assisted therapy (RT) vs Conventional therapy (physiotherapy, occupational therapy, task-oriented rehab, home program) | No significant superiority of RT over conventional therapy; outcomes were similar | 19 |  |
| **20** | Jingyi Wu, Hao Cheng, Jiaqi Zhang, Shanli Yang, Sufang Cai. | 2021 | Systematic review & meta-analysis | 41 RCTs, n = 1,916 stroke patients (mixed stages and severities). | Post-stroke upper-extremity motor impairment (hemiplegia). | Robot-assisted therapy (RT) — includes unilateral RT (URT) and bilateral RT (BRT); various robotic devices (end-effector and exoskeleton). Compared with dose-matched conventional therapist-led rehabilitation (same total treatment time). | Works (short term, small effect). RT produced a small but statistically significant improvement in FMA-UE immediately after intervention vs dose-matched conventional therapy; retention (follow-up) effect was not statistically significant. | 20 |  |
| **21** | Amirbekova M., Kispayeva T., Adomaviciene A., Eszhanova L., Bolshakova I., Ospanova Z. | 2025 | Systematic review & meta-analysis | 13 RCTs, n = 424 post-stroke patients (intervention n=216; control n=208); mean ages ranged ≈ 41.9–70 years. Studies included acute (n=4), subacute (n=6) and chronic (n=3) stages. | Stroke with motor impairments (upper and/or lower limb hemiparesis). | Robotic-assisted therapy (robotics) — a range of robotic devices (upper-limb robots, exoskeleton gloves, stationary gait trainers, wearable gait robots) compared with conventional rehabilitation / conventional physiotherapy (dose-matched standard care). Examples: ReHapticKnob, SEM™ Glove, REAplan®, Lokomat®, REX, Morning Walk, RoboGait. | Works (moderate benefit overall). Robotic therapy produced a moderate, statistically significant pooled effect vs conventional therapy (see Statistics row). Greatest effects in acute/subacute phases; limited effect in chronic phase. | 21 |  |
| **22** | Wu L., Xu G., Wu Q. | 2023 | Systematic review & meta-analysis | Adult stroke patients (total N≈709 across included RCTs). Mean age ≈58.4 yrs; 59% male. Time since stroke varied widely (median/mean ~13.7 months; range 16.1 days → 10.5 years) — so studies included subacute and chronic phases (not restricted to one phase). | Post-stroke lower-extremity impairment / limited walking ability. | Robot-Assisted Gait Training (RAGT) using the Lokomat® system, including Lokomat exoskeleton with body-weight support, adjustable guidance force and treadmill speed. (Robotic parameters reported: BWS commonly 30–50%; guidance force 20–100%; speed ~1 → 3 km/h). | Not superior overall to dose-matched conventional therapy across most outcomes; exception: balance (BBS) improved significantly with Lokomat. Conclusion in paper: Lokomat improves balance but otherwise shows no consistent superiority vs CPT. | 22 |  |

**Supplementary Table S2** Device Specifications, Biofeedback Modalities, and Integration Strategies

| **Device Type** | **Target_Extremity** | **Biofeedback** | **Integration Method** |
| --- | --- | --- | --- |
| Fixed exoskeleton robotic devices (mainly Lokomat® and gait trainer robot) | Lower limbs | Not explicitly reported; robotic devices provide kinematic feedback, but not EMG/visual biofeedback as intervention | RAGT alone or RAGT + CPT vs. CGT or therapist-assisted gait training |
| End-effector and Exoskeleton UL-RTs | Upper limb (proximal: shoulder–elbow, distal: wrist–hand) | Some devices integrated virtual reality/gaming or kinematic feedback (not consistent across all trials) | Assistive robotic training integrated with conventional therapy; some included trunk restraint, arm-weight support |
| Various: treadmill (with/without body-weight support), robotic ankle or gait devices, FES units, hippotherapy simulators, virtual/augmented-reality systems, vibration devices, etc. | Lower extremity (paretic and non-paretic limbs assessed) | Present in some categories (e.g., task-oriented biofeedback, visual feedback, auditory/rhythmic cueing) | Clinical physical-therapy interventions incorporating gait training, sensory cues, robotic assistance, or combined modalities |
| Non-wearable (treadmills, VR, force plates, cameras); Robotic assistive devices (exoskeletons, robotic arms, FES, cable-driven systems, KIINCE) | Mostly lower limb (paretic leg, feet); some upper limb (ankle joint EMG-FES) | Visual (25 studies), Auditory (13), Haptic (4), combined modes (11) | Real-time sensors (EMG, IMUs, force/pressure sensors, EEG, cameras, fNIRS, MRI) integrated with actuators for cues |
| Robotic exoskeletons (Lokomat, G-EO System, Walkbot, etc.) and end-effector gait robots | Lower limb | Mostly visual & proprioceptive (robot-provided gait correction), sometimes auditory | Robots integrated with physiotherapy programs (treadmill + harness, VR support, therapist-supervised) |
| Robotic exoskeletons (Lokomat, G-EO System, Walkbot, etc.) and end-effector gait robots | Lower limb | Mostly visual & proprioceptive (robot-provided gait correction), sometimes auditory | Robots integrated with physiotherapy programs (treadmill + harness, VR support, therapist-supervised) |
| Motorized treadmill, BWS harness systems, robotic orthosis, VR systems | Lower extremity | Present in VR and robotic-assisted gait training | Task-oriented repetitive training; VR integrates visual/auditory cues; robotics provide guided force |
| Lokomat, Walkbot, HAL, Ekso-GT, AutoAmbulator, SMA, BEAR-H1, MANBUZHE, GEMS, Exowalk, Welwalk, ExoAtlet Medy, curara® type 4, etc. | Lower extremity | Present in most robotic devices (e.g., auditory, kinematic, or visual cues) | Task-oriented, repetitive gait training; often paired with CPT focusing on gait, balance, and ADLs |
| Two main device classes reported: End-effector and Exoskeleton robots (various models used across trials). | Upper extremity (arm/hand). | Robots provided feedback/assistance during repetitive, task-oriented movements (paper discusses feedback as part of robotic training benefits). | High-repetition, high-intensity, task-oriented robotic training (some trials used robots to deliver/augment therapy in place of or in addition to standard therapist-led CR). |
| Exoskeleton robots (wearable and static, e.g., Lokomat, Ekso, SUBAR, RoboGait, robotic AFO) | Lower limb | Some devices included torque impedance/adaptive control, or integrated kinematic/step feedback (not universal) | Combined with treadmill training, overground training, or physiotherapy, depending on trial |
| Wearable powered exoskeletons (Ekso GT, EXOWALK, BEAR-H1, HAL, SMA, GEMS, AlterG Bionic Leg, etc.) | Lower limb (hip and/or knee, sometimes ankle) | Built-in sensors (accelerometers, gyroscopes, torque impedance, EMG signals); no universal feedback | Compared against dose-matched CGT (treadmill, overground, or physiotherapy programs) |
| Soft wearable robotic exosuit (ankle or hip-ankle assist) | Lower extremity | Not direct (no visual/auditory feedback), but devices provide assist-as-needed mechanical support with sensors for gait phase detection | Worn as a lightweight garment; provides powered ankle/hip assistance during gait training and overground walking |
| Upper limb rehabilitation robots: both end-effector and exoskeleton types (varied across RCTs) | Upper extremity | Present in most robotic systems (visual and/or haptic feedback during motor tasks) | Task-oriented repetitive training with robotic assistance, sometimes combined with therapist-guided conventional rehab |
| Robotic gait trainers (Lokomat and similar robotic exoskeleton systems) | Lower extremity | Present in most devices (visual, kinematic, or haptic gait feedback) | Task-oriented repetitive gait training with robotic assistance; integrated with CPT sessions |
| Mostly rigid exoskeletons (26/30), some soft exoskeletons (4/30); treadmill-based and overground devices (e.g., Lokomat, LOPES, other custom exoskeletons) | Lower limb (hip/knee/ankle — 1, 2 or 3 DoF depending on device) | Varies by study — many used kinematic/GRF-based signals; some included visual or proprioceptive feedback; EMG used as control input in few studie | Robots integrated with rehabilitation protocols (therapeutic or assistive scenarios); some studies used body-weight support, VR, or combined sensors |
| Robotic exoskeletons: Lokomat (static treadmill-based), Hybrid Assistive Limb (HAL) (overground, unilateral), Ekso (overground), Walkbot | Lower limbs | Proprioceptive input during limb loading; robotic control of joint motion allows near-normal sensory feedback | Robotic exoskeleton sessions combined with conventional physiotherapy or compared with conventional rehabilitation alone |
| Various robotic devices for upper-limb rehabilitation (end-effector and exoskeleton types) | Upper limb (shoulder, elbow, wrist, hand) | Real-time visual and/or haptic feedback from robotic interface | Robot-assisted training delivered alone or combined with standard physiotherapy/occupational therapy |
| Exoskeleton robots, End-effector robots | Lower limbs | Proprioceptive & sensory feedback emphasized; O-RAGT may provide stronger mechanoreceptor stimulation | Combined RAGT with conventional gait training in some studies; others used RAGT alone |
| Mixed: SMART Arm, Armeo Spring, REAplan, MIT-MANUS, NeReBot, Gloreha, Hand Mentor Pro, etc. | Upper limb (shoulder, elbow, wrist, hand, fingers) | Mostly kinematic feedback; some with conventional task feedback | RT delivered as stand-alone or adjunct to usual care |
| End-effector and exoskeleton devices (multiple makes/models across trials). End-effector devices showed significant pooled benefit; | Upper limb (shoulder/elbow ± forearm/wrist/hand) | Varied by device/trial; some robots were computer-enhanced or provided assist-as-needed control/feedback. Not standardized across studies. | Stand-alone robot training sessions delivered in trial protocols and dose-matched to conventional therapy (i.e., same therapist/contact time). Some trials combined robotic practice with usual therapy tasks; protocols varied. |
| Mixed: stationary treadmill/robotic gait trainers (Lokomat®, REAplan®, Morning Walk), wearable gait robots (curara®, REX), upper-limb robots and exoskeleton gloves (ReHapticKnob, SEM™ Glove, Hand of Hope), etc. | Upper limb and lower limb (both were included across trials). | Varied: some trials/devices incorporated VR/MR/interactive feedback, assist-as-needed control, or force/torque feedback; not uniform across studies. Combined robotics+VR or mirror therapy reported enhanced effects. | Robotic sessions delivered as the primary therapeutic modality in experimental arms, often integrated with routine physiotherapy tasks or adjuncts (VR, mirror therapy, cognitive tasks) in some trials. Controls received conventional therapy with similar dose/time. |
| Lokomat® robotic orthosis (exoskeleton for treadmill gait training; linear drives at hip & knee; BWS system). | Lower extremity / gait. | Device provides mechanized guided gait (robotic guidance + BWS); studies sometimes adjusted guidance force/parameters — explicit conventional biofeedback types not consistently reported across trials. | Lokomat used as the experimental training vs CPT. (In included trials Lokomat sessions replaced or supplemented usual gait training, but comparisons were performed against CPT typically matched for duration/frequency). |
| Mixed robotic devices (upper & lower limb; exoskeletons and end-effectors) | Both upper limb and lower limb (varied by included trials) | Mainly kinematic and visual feedback through robotic interface | RT delivered as stand-alone or adjunct to conventional therapy (dose-matched in most trials) |

**Supplementary Table S3** Conceptual Training Framework and Educational Modules for Robotic-Assisted Therapy in Stroke Rehabilitation

| **Basic Foundation** |  |  |
| --- | --- | --- |
| **Modul** | **Learning Outcome** | Topics |
| Human Anatomy & Physiology for Motion | Understanding human movement and related anatomy | 1. Neuromuscular Anatomy 2. Physiology of Neuromuscular 3. Basis of Kinematics 4. Basis of Motor Control |
| Biomechanics & Kinesiology | Understanding of Physical principles in human motion | 1. Force & Torque 2. Joint Mechanics 3. Basis of Gait & Posture |
| Neuroscience of Motor Control | Interpret Neuroanatomy and physiology into movement control mechanism | 1. Neuroplasticity 2. Motor Learning 3. Sensory Feedback |
| Introduction to Robotics & Mechatronics | Understand the Basic of Robotic concepts | 1. Sensors, Actuators, and Control systems 2. Basic of Robotics & Robotic rehabilitation technologies 3. Feedback Loops system (Biofeedback) |
| Programming Basics for Health Professional | Able to Inteprate health data | 1. Basic of Python Programming or MATLAB 2. Basis of Data Structure 3. Data Visualization |
| Rehabilitation Principles & evidence-Based Practice | Able to discuss about clinical principle and explain about evidance based practice in Stroke rehabilitation | 1. Basic of Research Methodologies 2. International rehabilitation standards (WHO ICF Model) 3. Treatment Design |
|  |  |  |
| **Intermediate Integration** |  |  |
| Stroke and Neurorehabilitation | Able to explain pathophysiology of stroke | 1. Stroke Pathophysiology and Classification 2. Neuroplasticity and Motor Recovery Stages 3. Principle of post-stroke motor learning |
| Assistive Robotics in Rehabilitation | Demonstrate of Robotics use in robotic rehabilitation technologies | 1. Hardware Components : actuators, sensors, joints, and structure 2. Motion control algorithms and user-robot interaction 3. Calibration and torque control' |
| Sensor & Biofeedback Systems | Understanding of device feedback information | 1. EMG & EEG signal recording setup and analysis 2. Pressure sensor setup and analysis 3. Haptic systems in rehabilitation |
| Data Acquisition & Analysis in Rehab Robotics | Analyze and organize Signal biomechanical data | 1. Signal Processing Analysis 2. Real-time Monitoring system 3. Outcome tracking 4. Patient data security |
| Human-Robot Interaction in Stroke Rehabilitation | Examine and experiment the safe and effective interaction in Stroke rehabilitation | 1. Synchronizing robotic assistance with physiological feedback 2. Adaptive control using bio-signals 3. Case study: EMG-control gait robots, EEG-control robots 4. Safety Standars |
| Clinical Protocol Design for Stroke Rehabilitation | Analyze and evaluate best protocol clinical trial design for Stroke | 1. Patient selection and contraindication 2. Session Plannning : Frequency, Intensity, Time, Techniques, Repetition 3. Integration with conventional therapy 4. Data intepretation and progress monitoring through quantitative assessment |
|  |  |  |
| **Advance Mastery - project based** |  |  |
| Advanced Human-Robot Interaction | Assemble or develop best practice model for user-specifics costumization in various stroke condition | 1. Adaptive learning algorithms for user-specific customization 2. Combination of multimodal feedback (EEG + EMG + Joint motion + Visual) 3. Adding Cognitive engagement in stroke robotic rehab |
| Machine Learning and AI in Rehabilitation Strategies | Develop effective and accurate AI-model for stroke rehabilitation | 1. Constructing Predictive modeling for gait/motor recovery outcomes 2. Real-time adaptive control using neural network algorithm 3. Annotation and model training in stroke rehabilitation datasets |
| Advanced Neurorehabilitation Strategies | Design a biofeedback-integrated rehabilitation protocol for post-stroke recovery | 1. Designing randomized controlled trials (RCTs) for stroke robotic rehab 2. Modifying exoskeleton control parameters for specific stroke patient needs 3. Ethics in technology-driven rehabilitation research 4. Cost-effective, sustanability, and technologies transfer & absorbtion |

Note : **Supplementary Table S3** outlines an authors-proposed conceptual training framework and learning modules for Robotic-Assisted Therapy (RAT) in stroke rehabilitation, designed to illustrate how such content could be integrated into clinical training pathways. This framework is theoretical in nature and does not correspond to any officially established or nationally accredited training program currently in use in Indonesia.

**Supplementary Table S4** presents a set of measurable parameters derived from key variables reported across the reviewed literature, structured as guiding questions to support systematic interpretation of acceptance of robotic-assisted technologies.

| **Perspective** | **Key Variable / Construct** | **Purpose** | **Question Item** | **Reference** |
| --- | --- | --- | --- | --- |
| **Patient perceptions** | Perceived Usefulness (PU) | assess the extent to which users believe that using the technology enhances their job performance. | 1. Using this technology improves my job performance. 2. Using this technology enhances my productivity. 3. This technology makes it easier to do my job. 4. I find this technology useful for my tasks. 5. This technology increases my effectiveness at work. | [Warkentin et al, 2007](https://www.researchgate.net/figure/Measurement-Items-for-Perceived-Usefulness-PU-and-Perceived-Ease-of-Use-PEOU_tbl2_220891511) |
|  | Perceived Ease of Use (PEOU) | evaluate how effortless users perceive the technology to be. | 1. I find this technology easy to use. 2. Learning to operate this technology is easy for me. 3. I find it easy to become skillful at using this technology. 4. I find this technology to be user-friendly. 5. I find this technology to be flexible to interact with. | [Lewis, 2019](https://www.researchgate.net/figure/The-Technology-Acceptance-Model-Davis-1989_fig1_340416809?utm_source=chatgpt.com) |
|  | Social Influence / Subjective Norms | measure the degree to which individuals perceive that important others believe they should use the technology. | 1. People whose opinions I value prefer that I use this technology. 2. In my circle of friends and colleagues, using this technology is common. 3. People important to me think I should use this technology. 4. Most people who are important to me use this technology. 5. People whose opinions I value have recommended this technology to me. | [Venkatesh et al., 2003](https://aisel.aisnet.org/misq/vol27/iss3/5/) |
|  | Attitude Toward Technology | assess users' overall affective reaction to using the technology. | 1. I enjoy using this technology. 2. I find this technology to be fun to use. 3. I feel comfortable using this technology. 4. I have a positive attitude toward using this technology. 5. I feel confident in my ability to use this technology. | [Davis, F. D., Bagozzi, R. P., & Warshaw, P. R. (1989).](https://pubsonline.informs.org/doi/fpi/10.1287/) |
|  | Digital / eHealth Literacy | evaluate the ability to seek, find, understand, and appraise health information from electronic sources. | 1. I know what health resources and information are available on the Internet. 2. I know how to find helpful health resources on the Internet. 3. I can use the Internet to answer my health questions. 4. I know how to use the Internet to find health information. 5. I am confident in my ability to use online health resources. | [van der Vaart & Drossaert, 2017](https://www.jmir.org/2017/1/e27/) |
| **Clinical perceptions** | Perceived Usefulness / Performance Expectancy | assess the perceived benefits of using the technology. | 1. Using this technology allows me to accomplish tasks more quickly. 2. Using this technology enhances my job performance. 3. I find this technology useful for my work. 4. This technology increases my productivity. 5. Using this technology improves my effectiveness at work. | [Gagnon et al., 2012](https://pubmed.ncbi.nlm.nih.gov/22082108/) |
|  | Perceived Ease of Use / Effort Expectancy | evaluate the ease of use associated with the technology. | 1. I find this technology easy to use. 2. Learning to operate this technology is easy for me. 3. I find it easy to become skilful at using this technology. 4. I find this technology to be user-friendly. 5. I find this technology to be flexible to interact with. | [Gagnon et al., 2012](https://pubmed.ncbi.nlm.nih.gov/22082108/) |
|  | Facilitating Conditions / Organizational Support | measure the resources and support available to use the technology. | 1. I have the resources necessary to use this technology. 2. My organization provides adequate support for using this technology. 3. I have the knowledge necessary to use this technology. 4. My organization has the infrastructure to support the use of this technology. 5. I have access to the necessary tools to use this technology. | [Gagnon et al., 2012](https://pubmed.ncbi.nlm.nih.gov/22082108/) |
|  | Workflow Compatibility / Time Efficiency | assess how well the technology fits into existing workflows and its impact on time efficiency. | 1. This technology fits well with my current work processes. 2. Using this technology saves me time. 3. This technology improves the efficiency of my work. 4. This technology integrates seamlessly into my workflow. 5. Using this technology reduces the time I spend on tasks. | [Greenhalgh et al., 2017](https://www.jmir.org/2017/11/e367/) |
|  | Professional Autonomy | evaluate the extent to which the technology allows professionals to exercise control over their work. | 1. This technology allows me to make independent decisions in my work. 2. Using this technology enhances my professional autonomy. 3. This technology supports my ability to work independently. 4. I feel more in control of my work when using this technology. 5. This technology enables me to exercise my professional judgment. | [Gagnon et al., 2012](https://pubmed.ncbi.nlm.nih.gov/22082108/) |
|  | Clinical Judgment | assess the ability to make informed decisions based on clinical expertise and patient data. | 1. This technology supports my clinical decision-making. 2. Using this technology improves my clinical judgment. 3. This technology provides valuable information for clinical decisions. 4. I trust the recommendations provided by this technology. 5. This technology enhances the quality of my clinical decisions. | [Greenhalgh et al., 2017](https://www.jmir.org/2017/11/e367/) |
|  | Trust in Technology’s Reliability and Safety | measure confidence in the technology's dependability and safety in clinical settings. | 1. I trust that this technology is reliable. 2. I trust that this technology is safe to use. 3. I have confidence in the performance of this technology. 4. I believe this technology operates without errors. 5. I trust that this technology will function as expected. | [Gagnon et al., 2012](https://pubmed.ncbi.nlm.nih.gov/22082108/) |

Reference Supplementary Table S4 :

1. Tedla JS, Dixit S, Gular K, Abohashrh M. Robotic-Assisted Gait Training Effect on Function and Gait Speed in Subacute and Chronic Stroke Population: A Systematic Review and Meta-Analysis of Randomized Controlled Trials. Eur Neurol. 2019;81(3-4):103-111. doi: 10.1159/000500747. Epub 2019 Jun 5. PMID: 31167193.
2. De Iaco L, Veerbeek JM, Ket JCF, Kwakkel G. Upper Limb Robots for Recovery of Motor Arm Function in Patients With Stroke: A Systematic Review and Meta-Analysis. Neurology. 2024 Jul 23;103(2):e209495. doi: 10.1212/WNL.0000000000209495. Epub 2024 Jun 13. PMID: 38870442.
3. Wonsetler EC, Bowden MG. A systematic review of mechanisms of gait speed change post-stroke. Part 1: spatiotemporal parameters and asymmetry ratios. Top Stroke Rehabil. 2017 Sep;24(6):435-446. doi: 10.1080/10749357.2017.1285746. Epub 2017 Feb 21. PMID: 28220715; PMCID: PMC5875693.
4. Pinheiro, C.; Figueiredo, J.; Cerqueira, J.; Santos, C.P. Robotic Biofeedback for Post-Stroke Gait Rehabilitation: A Scoping Review. Sensors 2022, 22, 7197. https://doi.org/10.3390/s22197197
5. Loro, A.; Borg, M.B.; Battaglia, M.; Amico, A.P.; Antenucci, R.; Benanti, P.; Bertoni, M.; Bissolotti, L.; Boldrini, P.; Bonaiuti, D.; et al. Balance Rehabilitation through Robot-Assisted Gait Training in Post-Stroke Patients: A Systematic Review and Meta-Analysis. Brain Sci. 2023, 13, 92. https://doi.org/10.3390/brainsci13010092
6. Lyu T, Yan K, Lyu J, Zhao X, Wang R, Zhang C, Liu M, Xiong C, Liu C, Wei Y. Comparative efficacy of gait training for balance outcomes in patients with stroke: A systematic review and network meta-analysis. Front Neurol. 2023 Apr 3;14:1093779. doi: 10.3389/fneur.2023.1093779. PMID: 37077566; PMCID: PMC10106590.
7. Yang J, Zhu Y, Li H, Wang K, Li D, Qi Q. Effect of robotic exoskeleton training on lower limb function, activity and participation in stroke patients: a systematic review and meta-analysis of randomized controlled trials. Front Neurol. 2024 Aug 13;15:1453781. doi: 10.3389/fneur.2024.1453781. PMID: 39193147; PMCID: PMC11347425.
8. Su, T., Wang, M., Chen, Z. and Feng, L. (2024), Effect of Upper Robot-Assisted Training on Upper Limb Motor, Daily Life Activities, and Muscular Tone in Patients With Stroke: A Systematic Review and Meta-Analysis. Brain Behav, 14: e70117. https://doi.org/10.1002/brb3.70117
9. Yang J, Gong Y, Yu L, Peng L, Cui Y, Huang H. Effect of exoskeleton robot-assisted training on gait function in chronic stroke survivors: a systematic review of randomised controlled trials. BMJ Open. 2023 Sep 14;13(9):e074481. doi: 10.1136/bmjopen-2023-074481. PMID: 37709309; PMCID: PMC10503387.
10. Hsu TH, Tsai CL, Chi JY, Hsu CY, Lin YN. Effect of wearable exoskeleton on post-stroke gait: A systematic review and meta-analysis. Ann Phys Rehabil Med. 2023 Feb;66(1):101674. doi: 10.1016/j.rehab.2022.101674. Epub 2022 Nov 30. PMID: 35525427.
11. Wu Y, Liu J, Md M, Zhao J, Wu S. Effectiveness of lower limb robotic rehabilitation on peak of oxygen uptake among patients with stroke: a systematic review and meta-analysis. BMJ Open. 2024 Oct 17;14(10):e082985. doi: 10.1136/bmjopen-2023-082985. PMID: 39419612; PMCID: PMC11487842.
12. Chuang, YC., Tsai, YL., Lin, T.TL. et al. Effects of soft robotic exosuit on ambulation ability in stroke patients: a systematic review. BioMed Eng OnLine 22, 88 (2023). https://doi.org/10.1186/s12938-023-01150-7
13. Carrillo, Cora, Tilley, Devyn, Horn, Kaitlyn, Gonzalez, Michelle, Coffman, Cassidy, Hilton, Claudia, Mani, Karthik, Effectiveness of Robotics in Stroke Rehabilitation to Accelerate Upper Extremity Function: Systematic Review, Occupational Therapy International, 2023, 7991765, 16 pages, 2023. https://doi.org/10.1155/2023/7991765
14. Hu, Mm., Wang, S., Wu, Cq. et al. Efficacy of robot-assisted gait training on lower extremity function in subacute stroke patients: a systematic review and meta-analysis. J NeuroEngineering Rehabil 21, 165 (2024). https://doi.org/10.1186/s12984-024-01463-1
15. Campagnini, S., Liuzzi, P., Mannini, A. et al. Effects of control strategies on gait in robot-assisted post-stroke lower limb rehabilitation: a systematic review. J NeuroEngineering Rehabil 19, 52 (2022). https://doi.org/10.1186/s12984-022-01031-5
16. Calafiore D, Negrini F, Tottoli N, Ferraro F, Ozyemisci-Taskiran O, de Sire A. Efficacy of robotic exoskeleton for gait rehabilitation in patients with subacute stroke : a systematic review. Eur J Phys Rehabil Med. 2022 Feb;58(1):1-8. doi: 10.23736/S1973-9087.21.06846-5. Epub 2021 Jul 12. PMID: 34247470; PMCID: PMC9980569.
17. Yang X, Shi X, Xue X, Deng Z. Efficacy of Robot-Assisted Training on Rehabilitation of Upper Limb Function in Patients With Stroke: A Systematic Review and Meta-analysis. Arch Phys Med Rehabil. 2023 Sep;104(9):1498-1513. doi: 10.1016/j.apmr.2023.02.004. Epub 2023 Mar 1. PMID: 36868494.
18. Chen S, Zhang W, Wang D, Chen Z. How robot-assisted gait training affects gait ability, balance and kinematic parameters after stroke: a systematic review and meta-analysis. Eur J Phys Rehabil Med. 2024 Jun;60(3):400-411. doi: 10.23736/S1973-9087.24.08354-0. Epub 2024 Apr 22. PMID: 38647534; PMCID: PMC11261306.
19. Chien WT, Chong YY, Tse MK, Chien CW, Cheng HY. Robot-assisted therapy for upper-limb rehabilitation in subacute stroke patients: A systematic review and meta-analysis. Brain Behav. 2020 Aug;10(8):e01742. doi: 10.1002/brb3.1742. Epub 2020 Jun 26. PMID: 32592282; PMCID: PMC7428503.
20. Wu J, Cheng H, Zhang J, Yang S, Cai S. Robot-Assisted Therapy for Upper Extremity Motor Impairment After Stroke: A Systematic Review and Meta-Analysis. Phys Ther. 2021 Apr 4;101(4):pzab010. doi: 10.1093/ptj/pzab010. PMID: 33454787.
21. Amirbekova M, Kispayeva T, Adomaviciene A, Eszhanova L, Bolshakova I, Ospanova Z. Systematic review and meta-analysis of effectiveness of robotic therapy in the recovery of motor functions after stroke. Front Hum Neurosci. 2025 Jul 21;19:1622661. doi: 10.3389/fnhum.2025.1622661. PMID: 40761519; PMCID: PMC12318999.
22. Wu L, Xu G, Wu Q. The effect of the Lokomat® robotic-orthosis system on lower extremity rehabilitation in patients with stroke: a systematic review and meta-analysis. Front Neurol. 2023 Dec 6;14:1260652. doi: 10.3389/fneur.2023.1260652. PMID: 38125828; PMCID: PMC10730677.
23. Warkentin, M., Johnston, A. C., & Shropshire, J. (2007). The IT security adoption conundrum: An initial step toward validation of applicable measures. *Proceedings of the 40th Annual Hawaii International Conference on System Sciences (HICSS 2007)*, 276–276. <https://doi.org/10.1109/HICSS.2007.59>
24. Lewis, J. R. (2019). Comparison of four TAM item formats: Effect of response option labels and order. *Journal of Usability Studies, 14*(4), 224–236. [https://uxpajournal.org/tam-formats-effect-response-labels-order/](https://uxpajournal.org/tam-formats-effect-response-labels-order/?utm_source=chatgpt.com)
25. Venkatesh, V., Morris, M. G., Davis, G. B., & Davis, F. D. (2003). MIS Quarterly, 27(3), 425–478. <https://doi.org/10.2307/30036540>
26. Davis, F. D., Bagozzi, R. P., & Warshaw, P. R. (1989). User acceptance of computer technology: A comparison of two theoretical models. *Management Science, 35*(8), 982–1003. <https://doi.org/10.1287/mnsc.35.8.982>
27. van der Vaart, R., & Drossaert, C. (2017). Development of the digital health literacy instrument: Measuring a broad spectrum of Health 1.0 and Health 2.0 skills. *Journal of Medical Internet Research, 19*(1), e27. <https://doi.org/10.2196/jmir.6709>
28. Greenhalgh, T., Wherton, J., Papoutsi, C., Lynch, J., Hughes, G., A’Court, C., Hinder, S., Fahy, N., Procter, R., & Shaw, S. (2017). Beyond adoption: A new framework for theorizing and evaluating nonadoption, abandonment, and challenges to the scale-up, spread, and sustainability of health and care technologies. *Journal of Medical Internet Research, 19*(11), e367. <https://doi.org/10.2196/jmir.8775>
29. Gagnon, M. P., Orruño, E., Asua, J., Abdeljelil, A. B., & Emparanza, J. (2012). Using a modified technology acceptance model to evaluate healthcare professionals' adoption of a new telemonitoring system. *Telemedicine journal and e-health : the official journal of the American Telemedicine Association*, *18*(1), 54–59. <https://doi.org/10.1089/tmj.2011.0066>
